# An autophagy-inducing and TLR-2 activating BCG vaccine induces a robust protection against tuberculosis in mice

**DOI:** 10.1038/s41541-019-0122-8

**Published:** 2019-08-05

**Authors:** Arshad Khan, Pearl Bakhru, Sankaralingam Saikolappan, Kishore Das, Emily Soudani, Christopher R. Singh, Jaymie L. Estrella, Dekai Zhang, Chandrashekhar Pasare, Yue Ma, Jianjun Sun, Jin Wang, Robert L. Hunter, N. Tony Eissa, Subramanian Dhandayuthapani, Chinnaswamy Jagannath

**Affiliations:** 1 Department of Pathology and Laboratory Medicine, University of Texas Health Sciences Center, Houston, TX USA; 2 Molecular and Translational Medicine, Paul L. Foster School of Medicine Texas Tech University Health Sciences Center, El Paso, TX USA; 3 Institute of Biosciences and Technology, Texas A&M University Health Science Center, Houston, TX USA; 4 Division of Immunobiology, Center for Inflammation and Tolerance, Cincinnati Children’s Hospital Medical Center, Cincinnati, OH 45229 USA; 5 Department of Biological Sciences and Border Biomedical Research Center, University of Texas at El Paso, Houston, TX USA; 6 Methodist Hospital Research Institute, Houston, TX USA; 7 Baylor College of Medicine, Houston, TX USA

**Keywords:** Live attenuated vaccines, Vaccines

## Abstract

*Mycobacterium bovis* BCG is widely used as a vaccine against tuberculosis due to *M. tuberculosis* (Mtb), which kills millions of people each year. BCG variably protects children, but not adults against tuberculosis. BCG evades phagosome maturation, autophagy, and reduces MHC-II expression of antigen-presenting cells (APCs) affecting T-cell activation. To bypass these defects, an autophagy-inducing, TLR-2 activating C5 peptide from Mtb-derived CFP-10 protein was overexpressed in BCG in combination with Ag85B. Recombinant BCG^85C5^ induced a robust MHC-II-dependent antigen presentation to CD4 T cells in vitro, and elicited stronger T_H_1 cytokines (IL-12, IL-1β, and TNFα) from APCs of C57Bl/6 mice increasing phosphorylation of p38MAPK and ERK. BCG^85C5^ also enhanced MHC-II surface expression of MΦs by inhibiting MARCH1 ubiquitin ligase that degrades MHC-II. BCG^85C5^ infected APCs from MyD88 or TLR-2 knockout mice showed decreased antigen presentation. Furthermore, BCG^85C5^ induced LC3-dependent autophagy in macrophages increasing antigen presentation. Consistent with in vitro effects, BCG^85C5^ markedly expanded both effector and central memory T cells in C57Bl/6 mice protecting them against both primary aerosol infection with Mtb and reinfection, but was less effective among TLR-2 knockout mice. Thus, BCG^85C5^ induces stronger and longer lasting immunity, and is better than BCG against tuberculosis of mice.

## Tuberculosis: Recombinant BCG vaccine shows enhanced protection via more efficient presentation

The BCG vaccine is widely used but has highly variable efficacy due at least in part to its inefficient processing by antigen-presenting cells (APC). Chinnaswamy Jagannath and colleagues at the University of Texas Health Sciences Center identify a peptide (C5) derived from the *Mycobacterium tuberculosis* (Mtb) virulence factor component CFP10 which can efficiently enhance BCG’s ability to activate APC function. C5’s activity in APCs is dependent on both Toll-like receptor 2 signaling and activation of autophagy which together enhances presentation of the Mtb protein Ag85B. A recombinant BCG vaccine over-expressing both Ag85B and C5 (BCG^85C5^) more strongly activates T_H_1-like responses which are known to be protective against Mtb infection. Mouse vaccination with BCG^85C5^ induces a qualitatively and quantitatively superior response to BCG—including greater expansion of Ag85B-specific T cells, more robust memory T cell formation and better control of Mtb in both lung and spleen.

## Introduction

*Mycobacterium tuberculosis* (Mtb) causes eight million new cases of tuberculosis and kills about two million people each year. HIV-1-induced CD4 depletion and emergence of multidrug-resistant (MDR) Mtb strains have aggravated the issue. *Bacille Calmette-Guérin* (BCG) is a widely used vaccine against tuberculosis, although meta-analysis of vaccination shows an unacceptably large variation in protective efficacy in children (0–80%), and again a variable efficacy against adult disease.^1^ BCG protected adults in certain geographic regions like the United Kingdom, while it seems to have failed in many developing countries. Its variable efficacy is multifactorial, including absence of the major immunogenic region of difference-1 (RD1) encoded antigens ESAT-6 and CFP-10 in BCG; variable expression of other antigens like MPT64 among sub-strains of BCG; and exposure of humans to environmental mycobacteria, thought to pre-sensitize and shift vaccine responses from T_H_1 to T_H_2 responses.^2^ Since BCG vaccine is safe, improving its immunogenicity appears to be a reasonable approach, although we and others have also generated Mtb-derived attenuated candidate vaccines.^3–5^ Many studies describe recombinant BCG strains with increased immunogenicity.^6^ Animal models indicate that both CD4 and CD8 T cells are important for immunity against tuberculosis.^7^ In humans, CD4 T cells appear to be critical since HIV-1-induced depletion leads to tuberculosis coinfection and increased death. On the other hand, CD8 T cells seem to contribute to long-term protection against tuberculosis in humans.

In our previous studies, we sought to determine the molecular basis of BCG-induced variable protection. Following vaccination, dendritic cells (DCs) and macrophages (MΦs) phagocytose BCG, process antigens within either proteasomes or lysosomes and present them, respectively, to activate CD8 and CD4 T cells. Interestingly, the standard mouse model for tuberculosis vaccination shows that protection correlates better with CD4 T cells than CD8 T cells.^7^ However, even among mice BCG generates only a modest protection against tuberculosis decreasing the lung burden of Mtb by about a log_10_. We, therefore, hypothesized that despite containing most antigens similar to Mtb, BCG is not efficiently processed and presented within antigen-presenting cells (APCs) like DCs and MΦs. This was strengthened by the suggestion that there were defects in antigen transfer between APCs and T cells.^8^ Our previous studies investigated the mechanisms of intracellular antigen processing and T cell activation to determine efficacy of the BCG and Mtb-derived vaccines. Non-pathogenic mycobacteria are taken up into phagosomes, which are sorted to fuse with lysosomes through a series of maturation events regulated by *rab* and SNARE proteins. However, both BCG vaccine and Mtb interfere with phagosome maturation through multiple mechanisms including the secretion of *sapM* phosphatase, which dephosphorylates phosphatidyl-inositol-3 kinase (PI-3K), a key initial trigger for phagosome maturation.^9–12^ We then demonstrated the molecular basis for decreased antigen presentation by BCG vaccine by demonstrating that Cathepsin-D cleaved secreted Ag85B and that sequestration of BCG in their near neutral pH phagosomes prevented efficient in situ digestion of Ag85B.^13^ We, therefore, established a direct link between “maturation arrest of BCG vaccine phagosomes and antigen presentation” particularly since Ag85B is an immunodominant component of Mtb that has been used frequently in subunit vaccines.

Interestingly, mammalian autophagy, which is regulated by a series of *Atg* genes, is another mechanism that delivers vaccines and pathogens to lysosomes, facilitating their degradation and antigen presentation. We reported the novel paradigm that inducing autophagy facilitates MHC-II-dependent mycobacterial antigen presentation to APCs,^14^ which was followed by the demonstration that autophagy-mediated MHC-I antigen presentation.^15^ In our study, rapamycin-induced autophagy improved delivery of BCG vaccine to lysosomes, better antigen presentation to both CD4 and CD8 T cells and markedly improved efficacy against tuberculosis in mice.^14^ Our discovery that the autophagy-inducing drug rapamycin increased the efficacy of BCG vaccine has been translated to human studies, as rapamycin enhanced the responses to flu vaccine among human elderly.^16^ Rapamycin is also a lead host-directed therapy agent to improve MΦ function during tuberculosis, although it also improves T-cell memory.^17^ In parallel with these studies on BCG, we also developed a lysosome-fusion competent mutant Mtb (*ΔfbpA*), which was more effective than BCG against tuberculosis in mice, and subsequently developed an *ΔfbpAΔsapM* candidate vaccine that enhanced the immunogenicity of MΦs in vitro and in mice.^18,19^

Although rapamycin enhanced the efficacy of BCG vaccine in mice, and that of flu vaccine in elderly humans, ethical considerations prevent its administration to infants. Thus in this study, we sought to improve the BCG vaccine through genetic engineering. Our earlier studies showed that overexpression of Ag85B in BCG (BCG^85B^; aka. rBCG30) triggered aggresome formation in APCs that triggered autophagy.^20^ Here, we explored whether we can genetically engineer a recombinant BCG vaccine capable of inducing autophagy.

Both Mtb and BCG secrete many immunogenic proteins, although BCG lacks the RD1 complex encoding ESAT-6 and CFP-10, which may, explain in part, its reduced immunogenicity in mice and humans.^21^ Indeed, ESAT-6 subunit vaccine provides protection against tuberculosis in mice comparable with BCG vaccine, and a recombinant BCG vaccine expressing ESAT-6 and Ag85B showed better protection than *wt*-BCG.^22–24^ Paradoxically, ESAT-6 inhibits IFN-γ responses of mouse and human T cells in vitro, also suppressing TLR-2-dependent phosphorylation of ERK in MΦs.^25–27^ Since ESAT-6 is secreted along with CFP-10 antigen by Mtb and form complexes at neutral pH, we sought to characterize the immunogenicity of this complex. We hypothesized that when ESAT-6 and CFP-10 complexes are internalized by APCs, their hidden domains may become separated in acidic pH of the endosomes, exposing their immunogenic and suppressive motifs. We demonstrate in this study that both CFP-10 and ESAT-6 proteins contain TLR-2 activating peptide motifs, one of which also induced autophagy. An autophagy-inducing, TLR-2 stimulating peptide (TSP) of CFP-10 was then co-expressed along with Ag85B in BCG to yield a novel BCG^85C5^ vaccine. BCG^85C5^ induced a stronger protection than *wt*-BCG against tuberculosis in mice, through expansion of CD62L^−^CCR7^−^CD44^hi^CD127^+/−^ effector T cells (T_EM_) and CD62L^+^CCR7^+/−^CD44^hi^ CD127^+^ central memory T cells (T_CM_), correlating with ≥1.5 log10 reduction in colony counts of Mtb in the lungs and spleens following infection or reinfection. Thus, autophagy induction and TLR-2 stimulation during BCG vaccination markedly improves its efficacy and longevity of protection through a combination of enhanced APC-T cell interaction and in vivo expansion of memory T cells.

## Results

### CFP-10 and ESAT-6 proteins differ in activating APCs in vitro

**Fig. 1:**
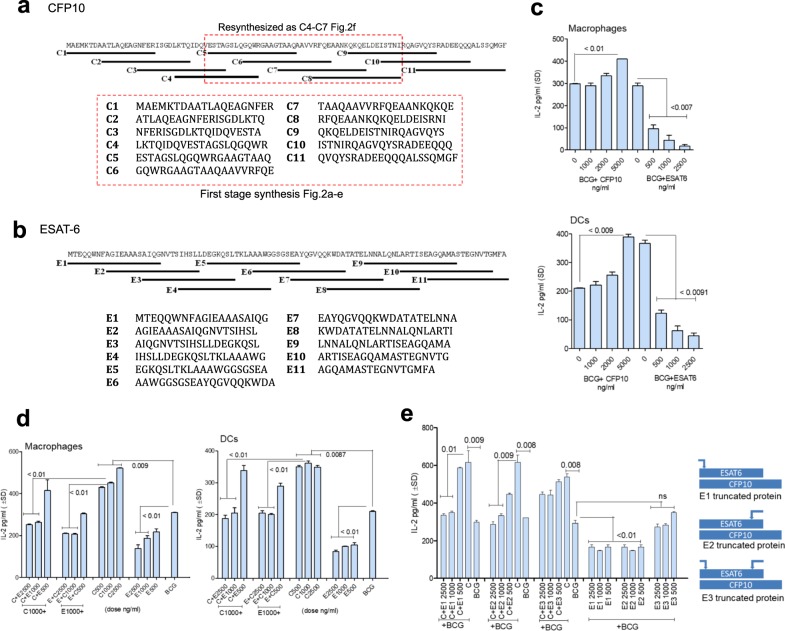
*Mycobacterium tuberculosis* (Mtb)-derived CFP-10 and ESAT-6 proteins show a differential activation of antigen-presenting cells in vitro. **a**, **b** Amino acid sequences of the whole proteins and overlapping peptides synthesized from CFP-10 and ESAT-6 are shown. CFP-10-derived first-stage synthesis peptides were subjected to an ELISA validation for their ability to stimulate antigen presentation (lower box), and “resynthesized” as peptides C4-C7 (upper box) and ELISA validation of the peptide designated as C5 in Fig. 2f. **c** C57Bl/6 mouse bone marrow-derived macrophages (MΦs) and dendritic cells (DCs) (APCs) were treated with recombinant CFP-10 and ESAT-6 whole proteins (BEI Resources Repository, NIH) at doses indicated, followed by infection with *Mycobacterium bovis* BCG (Pasteur; MOI = 1), and overlaid with antigen-85B-specific CD4 BB7 hybridoma T cells, for antigen presentation. The supernatants were tested for IL-2 levels using sandwich ELISA (one of three similar experiments shown; *p-*values, one-way ANOVA with Dunnett’s multiple comparisons post test). **d** A constant amount of CFP-10 (C; 0.5 µM; eq.ng/mL indicated) was mixed with ESAT-6 (E) in varying amounts (0.5–2.0 µM) in PBS at 37 °C for 15 min and added to MΦs, followed by infection with BCG and antigen presentation. CFP-10 and ESAT-6 were added alone as internal controls. Addition of ESAT-6 reduces the ability of CFP-10 to enhance in vitro antigen presentation (two experiments, *p-values, one-way ANOVA with Dunnett’s post test). **e** Three truncated proteins of ESAT-6 protein were prepared and added to CFP-10 protein with BCG vaccine, and antigen presentation determined using MΦs. The inhibitory motif of ESAT-6 lies near N-terminus (E1) and extends to middle (E2), but not at C-terminus (E3). Potential binding to CFP-10 is shown, which allows CFP-10 C-terminus to extend. Arrows above the ESAT-6 indicate the truncated regions

**Fig. 2:**
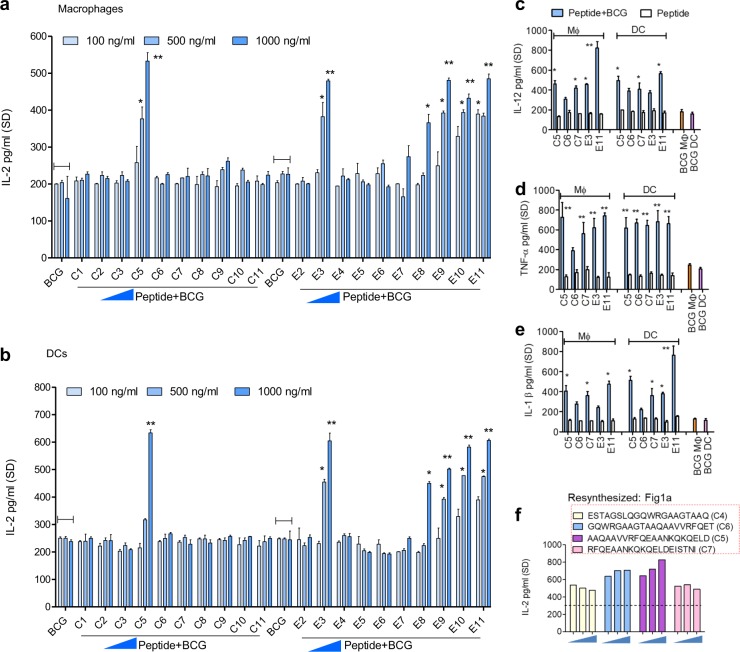
CFP-10 and ESAT-6 proteins contain immune-enhancing peptides, which activate antigen-presenting cells in vitro. Synthetic peptides derived from CFP-10 and ESAT-6 proteins were tested for their ability to enhance Ag85B epitope presentation by BCG infected APCs to CD4 BB7 T cells in vitro. **a**, **b** Distinct peptide moieties of CFP-10 and ESAT-6 stimulate antigen presentation (1000 ng/mL = 0.5 µM). Values are pg/mL ± SD from triplicate wells. **c–e** The stimulatory peptides moieties derived from CFP-10 or ESAT-6 (each tested at indicates doses) were combined with BCG to infect APCs for assay of T_H_1 pro-inflammatory cytokines in the supernatants using ELISA. **f** Peptides of CFP-10 were resynthesized to map “C5” peptide which elicited maximal antigen presentation from MΦs. All peptides of this panel show an increase in antigen presentation (*p* < 0.009 vs. BCG alone). Peptides were tested at indicates doses and dotted line represents IL-2 levels of BB7 T cells overlaid on BCG infected MΦs (*p < 0.01, **p < 0.003 vs. BCG alone control for all panels; one-way ANOVA with Dunnett’s post test*;* one of two similar experiments shown for each panel)

The protein sequence and overlapping peptides synthesized for CFP-10 and ESAT-6 are shown in Fig. 1a, b.^28^ Peptides were synthesized in two stages; those labeled C1–C11 were initially synthesized, and evaluated for their ability to stimulate APCs as indicated in Fig. 1c–e and Fig. 2a–f. Both CFP-10 and ESAT-6 antigens induce T cells and antibodies during tuberculosis of mice and humans, and are highly immunogenic. Paradoxically, ESAT-6 but not CFP-10 inhibits IFN-γ responses of mouse and human T cells in vitro.^27^ In addition, ESAT-6 suppresses MΦ signaling through TLR-2-dependent phosphorylation of ERK.^25–27^ Mtb secretes CFP-10 and ESAT-6 together, and they are thought to form a tight complex at neutral pH.^29,30^ We therefore examined the hypothesis that CFP-10 and ESAT-6 may exert individual effects, while their complexes have a different effect on APCs.^31–33^ We exploited the ability of APCs to process mycobacterial peptides and activate CD4 T cells via the MHC-II pathway to amplify T_H_1 immunity.^34^ An in vitro model of antigen presentation has been described where mouse APCs infected with Mtb or BCG vaccine present the peptide-25 epitope of secreted Ag85B to CD4 T cells (BB7 hybridoma).^13,35,36^ APCs were treated with CFP-10 and ESAT-6 proteins followed by BCG infection and overlaid with T cells to detect antigen presentation. Figure 1c illustrates that CFP-10 enhanced the ability of APCs to present antigen to CD4 T cells, whereas ESAT-6 showed an inhibition. A constant stimulatory dose of CFP-10 (around 0.5 µM) was then mixed with increasing amounts of ESAT-6 at 37 °C to enable complex formation, and incubated for 15 min prior to adding to APCs. ESAT-6 suppressed the stimulatory effects of CFP-10 on MΦs and DCs (Fig. 1d). A reverse combination was also tested. Since crystallographic studies suggest that CFP-10 and ESAT-6 form tight complexes at neutral pH,^29,37,38^ these data indicate that CFP-10 has the ability to stimulate APCs but its activity is likely blocked by complex formation with ESAT-6. In additional studies, three truncated peptides of ESAT-6 were prepared where the N- (E1), C- (E2), and both N- and C-termini (E3) were truncated. These were mixed with intact CFP-10 protein and added to MΦs for evaluation of antigen presentation. Figure 1e demonstrates that ESAT-6 lacking either N- or C-termini (E1; E2) still inhibited CFP-10 enhanced antigen presentation, while E3 failed to inhibit the stimulatory effect of CFP-10. Similar effects were observed for mouse bone marrow-derived DCs (unpublished observations).

### Peptide moieties of CFP-10 and ESAT-6 proteins are immune-enhancers for APCs

Although ESAT-6 suppresses mouse and human T-cell secretion of IFN-γ, it elicits strong T-cell responses in animal models and humans, and is therefore, a candidate vaccine antigen and indeed has been cloned into BCG.^39^ On the other hand, CFP-10 elicits robust T-cell responses in mice, reactive with T cells from humans with tuberculosis and does not have immune-suppressive activity.^40,41^ To explain the ambiguity in immunogenicity of these proteins, we proposed that their intracellular processing generated immunogenic moieties within APCs.

APCs were therefore, treated with synthetic overlapping peptides of these proteins followed by BCG infection, and in vitro antigen presentation as described previously. Figure 2a, b show two striking observations. The stimulatory activity of CFP-10 was localized to 50-70_aa_. In contrast, the intact ESAT-6 protein showed inhibitory activity (Fig. 1c). However, distinct peptides of ESAT-6, located both in the N-and the C-terminal region, strongly stimulated APCs to present Ag85B to BB7 T cells (Fig. 2a, b). None of the peptides elicited IL-2 from BB7 T cells overlaid on uninfected APCs (Supplementary Fig. S1). Furthermore, APCs treated with a combination of peptides with BCG, but not peptides alone at doses <2 µM, induced elevated levels of IL-12, TNFα and IL-1β, cytokines that boost T_H_1 immunity (Fig. 2c–f). In addition, the T_H_1 cytokine stimulating activity of CFP-10 and ESAT-6-derived peptides correlated with their ability to enhance antigen presentation (Fig. 2a, b vs. 2d, e) (Supplementary Fig. S2).

Since ESAT-6 protein inhibited antigen presentation by APCs (Fig. 1c), the CFP-10-derived immune-stimulatory peptides (red highlighted; Fig. 1a) were selected for further analysis. Overlapping peptides of the region 35-75_aa_ of CFP-10 were resynthesized and evaluated. A stretch of synthetic peptide named C5 (MW 2188.41, *Genscript*, USA; Fig. 2f; red highlighted) was identified as the optimal immune-stimulatory motif of the CFP-10 protein. It was then compared with E3 and E11 peptides and used for all studies described below.

### CFP-10 and ESAT-6-derived TLR-stimulating mycobacterial peptides (TSPs) regulate MHC-II-dependent antigen presentation and cytokine secretion through MyD88 and TLR-2

**Fig. 3:**
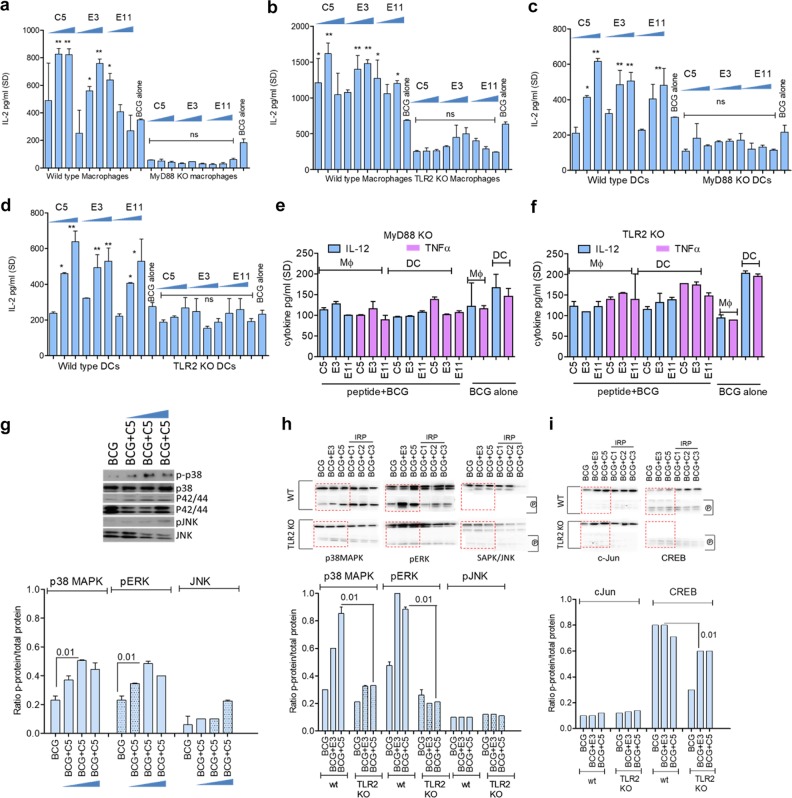
CFP-10 and ESAT-6-derived peptides activate APCs through MyD88 and TLR-2-dependent pathways. **a–d** APCs from wild-type C57Bl/6, MyD88, or TLR-2-deficient mice were tested using in vitro antigen presentation (peptides tested at 0.5, 1, and 2 µM; one of two similar experiments shown; values are mean of IL-2 concentration for triplicate wells; *p < 0.01, **p < 0.006 vs. BCG alone; one-way ANOVA). IL-2 levels secreted by BB7 T cells overlaid on knockout APCs are not significantly different. **e**, **f** APCs were activated or not with C5 and BCG and T_H_1 cytokines measured using sandwich ELISA. IL-12, and TNF-α cytokine responses are comparable with peptide activation followed by BCG vaccine infection or BCG infection alone among MyD88 or TLR-2 deficient APCs. **g** MΦs were infected with BCG or BCG with C5 peptide followed by western blot of lysates for phosphorylated MAPKs at 30 min. Densitometry shows that BCG + C5 peptide induce a stronger phosphorylation of p38 and ERK compared with native MAPK. Upper lanes show phosphorylated proteins, and lower lanes indicate whole protein. One of two similar experiments averaged for densitometry (*t* test). **h**, **i** MΦs from wild-type and TLR-2 knockout mice were incubated 4 h with C5 or E3 peptide or non-immunogenic peptides from CFP-10 protein followed by BCG infection for 90 min, followed by lysis and western blot assay for phosphorylated (P) MAPKs or c-Jun/AP-1 and CREB transcription factors. Densitometry (shown below blots) indicate that C5 peptide induces better phosphorylation in the presence of TLR-2 (phosphorylated bands marked (p); upper lanes have native proteins). One of two similar experiments is shown, which were averaged for densitometry (*t* test)

The mechanisms of action for TSPs were next investigated. Ag85B presentation assays were performed using MyD88 and TLR-2 knockout (TLR-2-KO) MΦs and DCs. The immune-enhancing activity of C5, E3, and E11 peptides was significantly decreased in MΦs and DCs of MyD88-KO and TLR-2-KO mice (Fig. 3a–d). Moreover, unlike wild type MΦs and DCs infected with TSP–BCG combination and illustrated in Fig. 2c–e, infection of the knockout APCs elicited comparable cytokine responses, emphasizing the pivotal role of MyD88 and TLR-2 during antigen presentation (Fig. 3e, f).

Of the three TSPs evaluated in the preceding studies (C5, E3, and E11), C5 peptide from CFP-10 was the most potent in enhancing in vitro Ag85B presentation (Fig. 2a, b) and inducing cytokine secretion (Fig. 2c–e), and was thus selected for additional studies. TLR-1/2 and TLR-2/6 signaling leads to the activation of IRAK-4/1 and TRAF-6, which in turn activates downstream MAP kinases (MAPK) and NF-kB (Supplementary Fig. S3).^42^ Phosphorylated MAPKs or activated p65 then activate AP-I and/or CREB transcription elements, which translocate to the nucleus. These events induce pro-inflammatory cytokine secretion and MHC-II production, which are essential for peptide epitope presentation to CD4 T cells. To confirm signaling through this pathway, MΦs were first infected with BCG with or without C5 peptide and lysates collected at intervals and analyzed using western blots. Figure 3g illustrates that C5 combined with BCG induced a stronger phosphorylation of both p38 and p42/44 ERK, whereas JNK was less significantly affected. In the next experiment, MΦs from *wt*-C57Bl/6 and TLR-2-KO mice were activated first with C5 or E3 peptide or non-stimulatory peptides from the N-terminus of CFP-10 protein (designated as C1, C2, and C3) for 4 h followed by BCG infection for 90 min, and lysates examined for phosphorylation. Quantitation of the blots confirmed that a combination of BCG with C5 enhanced the phosphorylation of p38 and ERK in *wt-*MΦs, which was reduced in TLR-2-KO MΦs (Fig. 3h, i).

### C5 enhanced surface expression of MHC-II in APCs is dependent on MAPK and AP-1/CREB

**Fig. 4:**
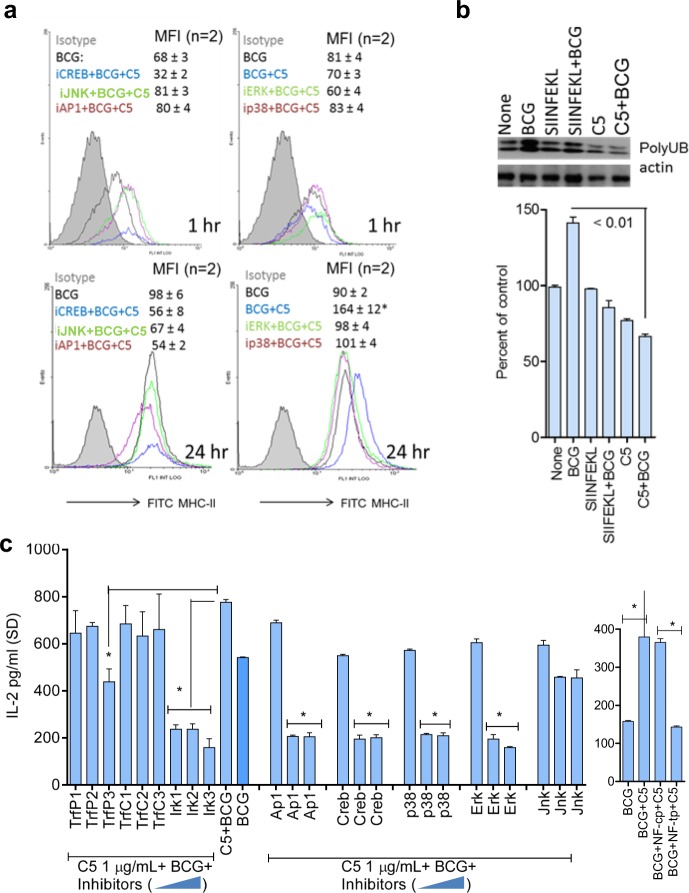
CFP-10-derived C5 peptide induces an upregulation of MHC-II in MΦs. **a** MΦs from wild-type C57Bl/6 mice were tested for surface expression of MHC-II. They were treated or not with 5 µM of inhibitors of API/CREB and MAPK, followed by 2 h activation with C5 peptide, and 1 or 24 h infection with BCG, followed by MHC-II staining and flow cytometry. Inset numbers indicate mean fluorescence intensity (MFI) values averaged from two experiments ( ± SD). C5 peptide enhances MHC-II expression in BCG-infected APCs and blockade of MAPK, AP-1/CREB reduces the levels of MHC-II (*p < 0.01; BCG + C5 group vs. inhibitors). **b** MΦs were tested naive or activated with C5 (2 h) and BCG (90 min) or irrelevant control SIINFEKL ova-peptide followed by BCG. Cell lysates prepared 4 h later were immune-precipitated with an antibody to MHC-II followed by probing with an antibody for ubiquitinated MHC-II. Densitometry (shown below) indicates that C5 activation decreases the levels of ubiquitinated MHC-II in MΦs, which is increased by BCG infection (two experiments ± SD; *t* test). **c** MΦs were incubated for 2 h with increasing doses of (0.5, 1, and 2 µM = lanes 1, 2, 3) specific (TrfP) or control (TrfP) peptide inhibitors of TRAF1/6; a peptide inhibitor of IRAK1/4 (IR1, 2, 3); peptide inhibitors of MAPKs; AP-1 and CREB and NF-kB (NF-tp, specific inhibitor; NF-cp, control peptide), followed by BCG or BCG + C5 for 2 h. Washed monolayers were overlaid with Ag85B-specific CD4 BB7 T cells, and IL-2 in the supernatant was measured after 18 h. Signaling blockade reduces antigen presentation by infected MΦs (*p < 0.009 vs. BCG alone; one-way ANOVA with Dunnett’s post test; one of two similar experiments shown)

BCG vaccine suppresses expression of MHC-II in phagocytic cells through the 19-kDa lipoprotein.^43^ Mycobacterial peptide loaded MHC-II is critical for activation of CD4 T cells, and we reported earlier that BCG-derived Ag85B peptide is less efficiently processed and presented to CD4 T cells by mouse MΦs.^13^ Although MΦs and DCs subtly differ in their ability to upregulate MHC-II, a common mechanism of upregulation of MHC-II is via the binding of the AP-1/CREB to the Class-II transactivator (CIITA) enhanceosome.^44,45^ To determine if C5 peptide affected antigen presentation through altering the levels of MHC-II, APCs were incubated with the inhibitors of MAPKs and AP-1/CREB, activated with C5 peptide followed by BCG infection and surface stained for MHC-II, before flow-cytometric analysis, as described before.^46^ Figure 4a indicates that C5 added to the BCG vaccine enhanced MHC-II expression over time in MΦs, which was significantly reduced when either MAPK or AP-1/CREB was inhibited. One mechanism that targets MHC-II for degradation is its ubiquitination through the enzyme MARCH1.^46^ To determine if C5 induced increase in MHC-II is due to prevention of its degradation, lysates of MΦs treated with C5 and BCG or a control (ovalbumin-derived) SINFEKL peptide with BCG were tested for ubiquitinated MHC-II using western blots as described.^46^ Figure 4b shows that C5 combination with BCG reduced the levels of ubiquitinated MHC-II compared with the control peptide. Since the latter is targeted for degradation through proteasomes, we propose that activation of MΦs with C5 peptide helps to maintain adequate levels of MHC-II for antigen presentation.^47^ Additional studies showed that C5 combination with BCG enhances MHC-II expression in DCs while, signaling inhibitors alone had no suppressive effect on BCG infected APCs (Supplementary Fig. S4). To further confirm the role of the signaling pathway that regulated MHC-II during antigen presentation, MΦs were incubated with specific and control peptide inhibitors of TRAF-6 and NF-kB; a specific peptide inhibitor of IRAK-4/1, and a previously validated set of known inhibitors of MAPK, C-jun AP-1, and CREB.^46^ MΦs were then activated with C5 peptide, followed by infection with BCG and antigen presentation. Antigen presentation was significantly reduced when these effectors were blocked with the exception of JNK, consistent with the profile of kinase phosphorylation from western blot data (Fig. 3g–h). It should be noted here that, lipoproteins and lipids of mycobacteria (19 kDa and LprG lipoproteins; lipoarabinomannan, LAM) cause multiple, mostly immuno-suppressive effects on APCs through TLR-2 and TLR-4-dependent pathways.^43,48,49^ Data shown above suggest that the C5 expressed by BCG can counteract the effects of these suppressive lipoproteins.

### C5 induces autophagy in MΦs increasing lysosomal delivery of the BCG vaccine and increasing antigen presentation

**Fig. 5:**
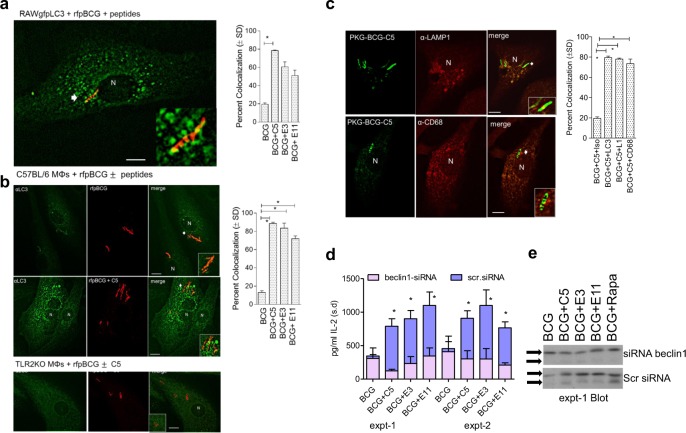
TLR-2 stimulating CFP-10-derived C5 peptide enhances autophagy in MΦs through LC3 binding. **a**, **b** RAW.A4 MΦs transfected with *gfp*LC3 were activated or not with TLR-2 stimulating peptides (TSPs) (1 µg/mL for 4 h), and infected with *rfp*-BCG and washed. Likewise, primary MΦs from either wt-C57Bl6 mice or TLR-2-KO mice were infected with *rfp*-BCG with or without TSPs, washed, fixed, and stained using antibodies to LC3 and imaged using confocal microscopy. Percent autophagosomes colocalizing with BCG were calculated from three separate experiments and plotted (*p < 0.009). *rfp*-BCG did not colocalize with LC3 in TLR-2 KO MΦs. C5 peptide alone had no effect on the numbers of autophagic puncta (not shown). **c** wt-C57Bl/6 MΦs were infected as above and stained for lysosome markers LAMP1 and CD68 followed by quantitation of labeling. **d** MΦs were either treated or not with siRNA vs. beclin1 to selectively block autophagy and scrambled siRNA as control, followed by infection with *wt*-BCG, or its combination with C5 peptide at a dose (1 µg/mL; 0.5 µM) known to enhance antigen presentation. BB7 CD4 T cells were overlaid on the MΦs, and IL-2 determined using sandwich ELISA. When autophagy was not blocked (blue fill), TSPs combined with BCG increased antigen presentation. When autophagy was inhibited using siRNA beclin1 (purple fill) antigen presentation was decreased (*p < 0.01 vs. scrambled siRNA control; two experiments, ± SD). *P-*values were determined using one-way ANOVA with Dunnett’s post test. **e** Lysates of MΦs from one of the ELISA experiments were analyzed using an antibody to LC3; they show reduced lipidation of LC3 bands after siRNA vs. beclin1 treatment

BCG is an interesting live attenuated vaccine that sequesters in immature phagosomes of MΦs, and we demonstrated earlier that maturation arrest interferes with antigen presentation.^13^ Thus, MΦs infected with BCG were less able to present the p25 epitope from Ag85B to CD4 cell hybridoma in vitro.^13^ On the other hand, we demonstrated that rapamycin-induced autophagy in BCG-infected MΦs and DCs increased antigen presentation to CD4 T cells.^14^ Interestingly, TLR-2 ligands are known to facilitate a unique type of uptake and lysosomal localization of particulate antigens involving the microtubule-associated light chain-3 (LC3), which is also a marker of the autophagosome.^50,51^ To determine whether C5 peptide induced TLR-2 activation and enhanced LC3 labeling and thus autophagy, RAW-MΦs transfected with *gfp*LC3 were infected with *rfp*-tagged BCG mixed with C5, E3, and E11 peptides. Following phagocytosis and wash, LC3 colocalization was evaluated using the Nikon metaview software. Figure 5a illustrates that C5 peptide markedly enhanced the colocalization of *gfp*LC3 with *rfp*-BCG. Since RAW-MΦs are from BALB/c mice, and not suitable to demonstrate a correlation between autophagy and antigen presentation, in the next experiment, C57Bl/6 mouse bone marrow-derived primary MΦs were then infected with *rfp*-BCG mixed with TSPs, incubated for 24 h and stained for LC3 using antibodies and FITC-anti IgG conjugates. Figure 5b shows that *rfp*-BCG mixed with the TSPs showed enhanced labeling with LC3 confirming autophagosome formation. Interestingly, MΦs derived from TLR-2 KO mice showed poor staining of LC3 on *rfp*-BCG phagosomes, suggesting that C5 induced autophagy through TLR-2. Finally, C5 enhanced the colocalization of *gfp*BCG phagosomes with LAMP1 and CD68, which are two markers of lysosomes (Fig. 5c).

TLR-2 activation initiates a unique kind of LC3-dependent autophagy among mouse and human APCs, facilitating an MHC-II-dependent antigen presentation.^50,51^ Since C5 peptide enhanced LC3 labeling and lysosomal localization through TLR-2, *wt*-MΦs were treated with siRNA vs. beclin1 to knockdown autophagy, infected with BCG with or without C5 peptide and antigen presentation was evaluated. Figure 5d illustrates that siRNA KO significantly decreased antigen presentation even when BCG was treated with C5 peptide. In addition, the lysates of such MΦs were found to have decreased lipidation of LC3 confirming autophagy knockdown (Fig. 5e). These data together support the observation that C5 peptide enhances autophagy and lysosomal localization for BCG vaccine, thereby increasing the MHC-II-dependent presentation of Ag85B-derived peptide.

### C5 peptide mixed with BCG protects against aerosol-induced tuberculosis in mice

Since C5 peptide enhanced in vitro immunogenicity of BCG-infected MΦs, we sought to determine its in vivo effect. A series of CFP-10 peptides including C5 and selected ESAT-6-derived peptides were mixed with BCG, and mice were vaccinated followed by aerosol challenge with Mtb. The data show that among the CFP-10 peptides, only C5 enhanced the protective efficacy of BCG vaccine when given at as two small doses with BCG (Supplementary Fig. S5). In contrast, among ESAT-6-derived peptides, E1 and E2 nullified the effect of BCG while E3, E10, and E11 had no significant booster effect on BCG. These observations correlate with the ability of ESAT-6 to inhibit MHC-II expression in MΦs and T-cell responses.^27,52^

### Genetic engineering of TLR-2 stimulating peptide C5 into BCG vaccine and validation

The data presented above indicated that the C5 peptide exerted a novel “adjuvant-action” on BCG infected APCs, which translated into increase in the efficacy of CFP-10 peptides mixed with BCG vaccine. C5 was also superior to E3 and E11 peptides (Supplementary Fig. S5). We reported earlier that, overexpression of Ag85B in BCG Pasteur (creating BCG^85B^; aka. rBCG30) enables secretion of Ag85B in copious quantities, which induces aggresome-dependent autophagy and enhances efficacy against tuberculosis in mice.^14^ Since rBCG30 ( = BCG^85B^) has undergone human trials, and could potentially replace *wt-*BCG^53,54^; we sought to further increase the efficacy of the BCG^85B^ vaccine through expression of C5 peptide.

C5 peptide was expressed (methods) as a part of secreted Ag85B of BCG^85B^ vaccine yielding BCG^85C5^. For comparison, the whole CFP-10 protein was expressed along with Ag85B yielding BCG^85CFP^. In vitro antigen presentation studies confirmed that BCG^85C5^ showed stronger and sustained antigen presentation in mouse DCs over 5 days compared with *wt*-BCG (Supplementary Fig. S6). Importantly, BCG^85C5^ elicited an Ag85B epitope presentation to CD4 T cells from human cord blood-derived MΦs, which was significantly decreased after TLR-2 receptor knockdown (Supplementary Fig. S7). BCG^85C5^ elicited a robust reactive oxygen species response from *wt-*MΦs, which decreased in TLR2R-KO MΦs, confirming its ability to activate TLR-2 (Supplementary Fig. S8). BCG^85C5^ also elicited a strong antigen presentation from bone marrow-derived DCs of wt-C57Bl/6 mice, which was markedly decreased among DCs with a conditional knockout of *atg7* on C57Bl/6 background (Supplementary Fig. S9). Finally, when splenocytes from BCG^85C5^ or other BCG strains were incubated in vitro with recombinant CFP-10 protein, only those from BCG^85C5^ vaccinated mice elicited robust IFN-γ response (Supplementary Fig. S10).

The parent BCG^85B^ was then compared with BCG^85C5^ vaccine using *wt-* and TLR-2 KO mice to determine if TLR-2 was required for the efficacy of BCG^85C5^ against tuberculosis challenge of mice. In a second and expanded study, *wt-*C57Bl/6 mice were used to compare the efficacy of BCG^85C5^ with BCG^85CFP^ for memory T-cell responses and protection against tuberculosis. It should be noted here that both these constructs overexpress Ag85B.

### BCG^85C5^ vaccine requires TLR-2 for better vaccine efficacy in mice compared with *wt-*BCG or BCG^85B^ vaccine

In the well-established NIH mouse model of tuberculosis vaccine evaluation, *wt-*BCG protects against aerosol-induced tuberculosis reducing the lung colony (CFU) counts of Mtb by a log_10_ 4 weeks after challenge.^55^

#### Post-vaccination immune responses

**Fig. 6:**
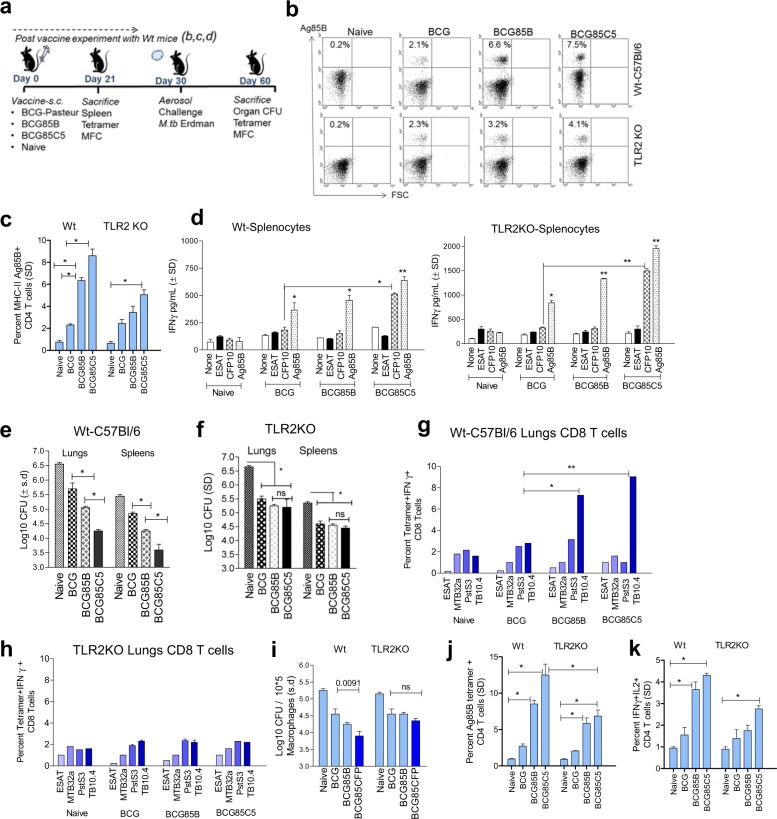
Recombinant BCG vaccine expressing Ag85B and C5 peptide (BCG^85C5^) induces TLR-2-dependent protection against aerosol-induced tuberculosis of mice. **a** NIH short-term BCG vaccine induced protection mouse model. C57Bl/6 mice (4–6 weeks M/F) were vaccinated as indicated with BCG strains or left untreated (naive); followed by aerosol challenge with 100 CFU per mouse of virulent *M. tuberculosis* Erdman (Mtb) and killed for bacterial (CFU) counts of lungs and spleens and for T-ell assays. **b**, **c** Splenocytes on day 21 were stained using Ag85B-MHC-II tetramer (NIH tetramer core; Emory University) for CD4 T cells. The data averaged for three mice; *t* test. **d** Splenocytes were cultured with soluble antigens (5 µg/mL) as indicated for 18 h and IFN-γ ELISA. The data averaged for three mice per group (mean ± SD; *p < 0.01; **p < 0.0071; one-way ANOVA). **e**, **f** On day 60, organs were harvested for Mtb counts. BCG^85C5^ yields better protection than *wt-*BCG and BCG^85^ in reducing the load of Mtb in the lungs and spleens of wild-type mice. Protection generated by BCG^85C5^ is significantly reduced in TLR-2 knockout mice (*p < 0.0091; two-way ANOVA; *n* = 5 mice per group and vaccine). **g**, **h** Lung T cells of wild-type and TLR-2 knockout mice on day 60 (*n* = 3 per group) were analyzed with flow cytometry using tetramers specific for MHC-I type epitopes from ESAT-6, Mtb32a, PstS3, and TB10.4 antigens of Mtb (*histograms illustrated in* Supplementary Fig. S11) (see the Methods section). Cells were gated on CD8, intracellular IFN-γ and MHC class-I tetramers. BCG^85C5^ induces a stronger expansion of antigen-specific tetramer-positive CD8 T cells in wild-type mice compared to TLR-2 knockout mice (*p < 0.01; one-way ANOVA). **i** Purified CD8 T-cell pools from lungs of vaccinated or naive mice (*n* = 3 per group) were overlaid on Mtb infected macrophage monolayers followed by CFU counts at 72 h (*p-*values, vaccine groups vs. naive; one-way ANOVA). **j**, **k** Lung T cells of mice collected on day 60 (*n* = 3 per group) were stained using Ag85B-specific MHC-II tetramer and for multifunctional cytokine secreting CD4 T cells (MFCs) (*p < 0.01). *P-*values were determined using one- or two-way ANOVA with Dunnett’s post-test

The NIH mouse model with *wt-*C57Bl/6 and TLR-2-KO mice were used to compare the efficacy of *wt-*BCG Pasteur with BCG^85B^ and BCG^85C5^ (Fig. 6a). To ascertain immunogenicity of the vaccines, 21 days after vaccination, mice were killed and splenocytes analyzed for MHC-II Ag85B-specific CD4 T cells (NIH core tetramer facility, Emory University, Methods) and in vitro recall immune responses to antigens.^56^ The data show that, BCG^85C5^ induced a twofold expansion of antigen-85B-specific CD4 T-cell responses post vaccination among *wt-*C57Bl/6 compared with TLR-2-KO mice (Fig. 6b–c). In addition, IFN-γ levels of the supernatants of splenocytes activated with four individual antigens confirmed that BCG^85C5^ vaccinated T cells from *wt-*C57Bl/6 showed twofold stronger recall responses (Fig. 6d). The antigens were selected such that recall responses could occur in a specific manner; thus, Ag85B was present in both BCG and Mtb whereas ESAT-6 and CFP-10 were absent in BCG and present in Mtb. Figure 6d for example, illustrates that, CFP-10 elicited IFN-γ recall responses in splenocytes of mice given only BCG^85C5^, but not those given either wt-BCG or BCG^85B^. These data support the observation that BCG^85C5^ enhances both in vitro and in vivo antigen presentation and expands Ag85B-specific CD4 T cells.

#### Post-aerosol challenge immune responses

Since the specificity and immunogenicity of BCG^85C5^ was established, *wt-*C57Bl/6 mice and TLR-2-KO mice were used to evaluate vaccine efficacy. Protection against tuberculosis challenge was correlated with levels of CD4 and CD8 T cells important for defense against tuberculosis. Figure 6e illustrates that BCG^85C5^ generated better protection than *wt-*BCG or BCG^85B^ vaccine in the lungs and spleens of *wt-*C57BL/6 mice. Interestingly, protection generated by BCG^85C5^ was significantly reduced in the organs of TLR-2-KO mice, underscoring the importance of TLR-2 (Fig. 6f). BCG^85B^ induces robust CD4 and CD8 T-cell responses in mice.^14^ To determine the antigen specificity of BCG-induced T cells mediating protection against tuberculosis, T cells were phenotyped using three well-characterized CD8 tetramers along with an MHC-II tetramer for Ag85B (NIH core tetramer facility, Emory University, USA). Of these, TB10.4 antigen elicits robust response in mice. BCG^85C5^ induced significantly higher levels of IFN-γ^+^ CD8 T cells specific for TB10.4 antigen of Mtb in the lungs (Fig. 6g; *Histograms illustrated in* Supplementary Fig. S11). Both TB10.4 and other antigen-specific CD8 T cells were reduced in the lungs of TLR-2-KO mice (Fig. 6h). Bead purified CD8 T cells from the lungs of these mice were then evaluated for ability to kill Mtb in vitro, and they were functionally active (Fig. 6i). Since TB10.4^+^ CD8 T cells are associated with protection against tuberculosis in mice, these data confirm the superior efficacy of BCG^85C5^ and its dependence on TLR-2.^57^ Finally, the lungs of vaccinated mice contained elevated levels of Ag85B-specific CD4 T cells and MFCs (Fig. 6j, k). These data are consistent with the observation that both Mtb infection and BCG vaccination expand Ag85B-specific CD4 T cells in vivo, and unlike *wt-*BCG, BCG^85C5^ induces better responses of MFCs in mice.^56,58^^,59^

### Primary and reinfection tuberculosis models in *wt-*C57Bl/6 mice to measure the expansion of T_EM_ effector and T_CM_ memory T cells correlating with protection

**Fig. 7:**
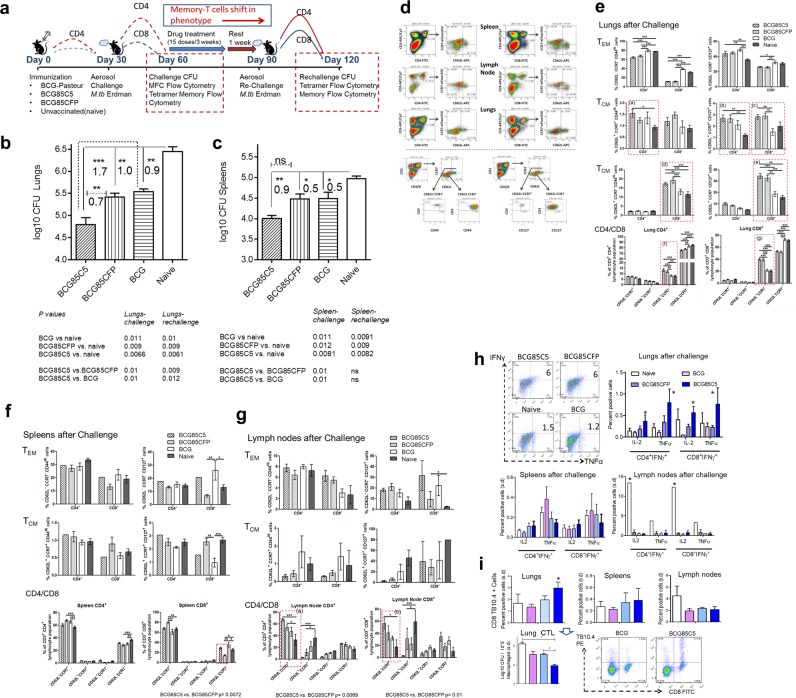
BCG^85C5^ vaccine induces a robust protection against primary challenge of tuberculosis in mice through effector (T_EM_) and central memory (T_CM_) T cells. a NIH short-term protection (Fig. 6a) and a long-term protection (rechallenge) models are shown. Rechallenged mice were vaccinated, treated with isoniazid and rifampin (mix of 10 mg/kg dose each), rested and challenged with Mtb. The data for NIH and rechallenge models are shown in Fig. 7 and Fig. 8, respectively. Post-vaccine mice were killed on day 21, post challenge on day 60 and post rechallenge mice on day 120. Post vaccination Ag85B-specific T cell analysis in Supplementary Fig. S10. **b**, **c** CFU counts of Mtb in the lungs and spleens after primary challenge (day 60). BCG^85C5^ reduces Mtb CFUs (mean ± SD) of lungs and spleens better than BCG. *P-*values and log10 difference of CFUs indicated (footnote; *n* = 5 mice per group; two-way ANOVA). **d** Histogram analysis of T cells for memory markers using flow cytometry. **e**–**g** BCG^85C5^ induces both T_EM_ and T_CM_ T cells in the lungs after primary challenge. Vaccine groups with significant differences in memory populations are highlighted (*p < 0.02; **p < 0.01; ***p < 0.008; one-way ANOVA). BCG^85C5^, BCG^85CFP^ and *wt-*BCG induce comparable numbers of T_EM_ in lungs, spleens, and lymph nodes, while, BCG^85C5^ and BCG^85CFP^ induce better T_CM_ than *wt-*BCG in the target organ lungs (Fig. 7e; p boxes **a**–**e**). CD4/CD8 panels (Fig. 7e, f, g) indicate percent of cells calculated after estimating absolute numbers (*n* = 3 mice), which were then averaged. BCG^85C5^ increases absolute numbers of effector CD62L^−^ CD4 and CD8 T cells in the lymph nodes compared with wt- BCG (vs. Fig. 7g; p boxes **a**, **b**) and effector CD62L^−^ CD8 T cells in the spleens (vs. Fig. 7f). **h** BCG^85C5^ enhances levels of multifunctional cytokine secreting T cells of lungs (*p < 0.01; one-way ANOVA). **i** TB10.4 tetramer specific CD8 T cells of the lungs, spleens, and lymph nodes. Clockwise. TB10.4^+^ CD8 T cells in naive or vaccinated mice. Histogram of TB10.4^+^ CD8 T cells. Purified CD8 pools (*n* = 3) of naive or vaccinated group, overlaid in vitro on Mtb-infected macrophages followed by CFU counts. (*p < 0.009; one-way ANOVA). *P-*values determined using one- or two-way ANOVA with Dunnett’s post-test

The BCG vaccine induces significant CD4, but reduced CD8 cell responses in mice which correlates with short-term protection against tuberculosis in mice.^60^ T cells expressing memory markers have been demonstrated in the lungs of mice following BCG vaccination.^61^ Some investigators have suggested that decreased long-term protection in mice could be due to the lack of cells mediating central memory.^62^ Using a mouse reinfection model of tuberculosis, CD4 T cells expressing memory phenotype were found to generate a transient protection against tuberculosis.^62^ BCG^85^ vaccine mixed with C5 peptide activated APCs to induce robust TH1 cytokines and antigen presentation to CD4 T cells in vitro through enhanced surface expression of MHC-II. Figure 6 confirmed that TLR-2 mediated activation of APCs translated well into better protection against aerosol-induced tuberculosis of mice. Thus, we sought to examine the ability of BCG^85C5^ to generate T_EM_ and T_CM_ using an improved tuberculosis rechallenge mouse model. In this model illustrated in Fig. 7a, the first half represented the NIH model (primary challenge model), and the second half, allowed vaccine-induced T_EM_ to expand in response to a challenge with tuberculosis, followed by a drug-induced cure of infection, and a resting period which facilitated the emergence T_CM_.^55,57,63^ Mice were then rechallenged with Mtb (aka. reinfection model) to determine whether T_CM_ could protect against tuberculosis.

Since BCG^85C5^ was found superior to BCG in Fig. 7, in this experiment BCG^85C5^ was compared with BCG^85CFP^ and *wt-*BCG. Thus, *wt-*C57Bl/6 mice were vaccinated with *wt-*BCG, BCG^85CFP^, and BCG^85C5^ followed by aerosol infection at 4 weeks (day 60) (Fig. 7a). Post-vaccination and post-challenge immune responses were evaluated as in Fig. 7 and correlated with protection enumerating Mtb CFUs on day 60. A duplicate set of mice vaccinated and challenged as above were treated with drugs (Isoniazid and rifampin) to clear both vaccine and Mtb organisms, followed by a resting period and rechallenge with virulent Mtb on day 90 (Fig. 7a). Post-rechallenge immune responses were correlated with protection against reinfection (log_10_ reduction in CFUs) determined by Mtb CFU counts 30 days after rechallenge (Fig. 7a). Several earlier studies assessing protection against viral and bacterial pathogens of the mouse model indicate that T_EM_ cells are phenotypically CD62L^−^CCR7^-^CD44^hi^CD127^+/−^ whereas, T_CM_ memory T cells are CD62L^+^CCR7^+/−^CD44^hi^ CD127^+^.^62^^,64–67^ Based on receptor distribution, two additional subsets of T_EM_ and four subsets of T_CM_ were typed using vaccinated and naive mice with optimized markers and flow-cytometry data presented as percent distribution and absolute numbers of CD4 and CD8 memory T cells per organ.

### BCG^85C5^ vaccine induces stronger T_EM_ effector and T_CM_ central memory cells which correlate with better “short-term protection” against aerosol-induced tuberculosis

Post-vaccination experiments performed on day 21 (Fig. 7a) confirmed that BCG^85C5^ and BCG^85CFP^ were markedly immunogenic in mice expanding the Ag85B-specific CD4 T cells (Supplementary Fig. S12). Figure 7b, c demonstrate that BCG^85C5^ generated better protection in lungs and spleens compared with both *wt-*BCG and BCG^85CFP^ after primary challenge. Statistical evaluations confirmed that BCG^85C5^ is superior to *wt*-BCG or BCG^85CFP^ in reducing Mtb counts of lungs (footnote, Fig. 7b, c). On day 60, three additional mice per group were individually analyzed for T cells using flow cytometry; a typical analysis is shown in Fig. 7d.

#### Role of CD4 T_EM_ vs. T_CM_

Since CD4-deficient mice are extremely susceptible to tuberculosis, it is likely that they perform a protective function, although contribution by other immune cells cannot be ruled out.^68,69^ Consistent with this observation, all three BCG vaccines induced robust CD62L^−^ T_EM_ CD4 T-cell responses in the lungs, spleens, and lymph nodes (Fig. 7e, f, g). Interestingly, BCG^85C5^ and BCG^85CFP^ induced better expansion of CD62L^+^ T_CM_ CD4 T cells in the lungs (p groups, a, b, c, d, e*;* Fig. 7e). However, BCG^85C5^ was better than BCG^85CFP^ and *wt*-BCG in generating more CD62L^−^ T_EM_ CD4 T cells in the lymph nodes (p group a, b*;* Fig. 7g). Since adaptive immune responses in mice depend on mycobacterial antigen production in lymph nodes, these data support the concept that BCG^85C5^ is more immunogenic than either BCG^85CFP^ or *wt-*BCG.^59^

#### Role of CD8 T_EM_ vs. T_CM_

*wt-*BCG is a poor inducer of CD8 T cells in mice and cloning of a pore-forming toxin listeriolysin (LLO) enhances the ability of BCG^LLO^ to induce CD8 T cells.^70^ On the other hand, Mtb infection of mice induces CD8 T cells and prior BCG vaccination can increase their numbers.^71^ Consistent with these observations, all four groups of challenged and vaccinated mice had elevated levels of CD8 T_EM_ in the lungs (Fig. 7e). However, the effects of prior vaccination were striking. Thus, both BCG^85C5^ and BCG^85CFP^ increased the CD62L^+^ subsets of CD8 T_CM_ cells in the lungs compared with mice given *wt-*BCG and naive mice which experienced only Mtb infection (p groups, c, d, e, g; Fig. 7e). BCG vaccinated mice show a dominance of effector T cells in the lungs.^62^ In this study, all vaccines maintained elevated levels of T-cell effectors in the lungs, although BCG^85C5^ increased CD62L^-^ CD8 T_EM_ in the lymph nodes ^and^ was superior to BCG^85CFP^ and BCG (p group b, Fig. 7f). Mice chronically infected with Mtb show a dominance of effector over central memory T cells with a reverse profile in the lymphoid organs.^57^ This study shows that, unlike *wt-*BCG vaccinated or Mtb infected mice, recombinant BCG^85C5^ and BCG^85CFP^ induce a robust mix of T_EM_ and T_CM_ in the lungs correlating with better protection against bacterial growth. We propose that BCG^85C5^ is better than BCG^85CFP^ in generating protection (footnote, Fig. 7b, c; log differences in killing Mtb is also shown) because it induces more functional effectors in the lungs and lymph nodes (Fig. 7e–g).

### BCG^85C5^ vaccine induces better multifunctional T cells and functional CD8 T cells in mice

Cytokine secreting T cells are mediators of T_H_1 immunity and MFCs mediate better immunity against tuberculosis among mice given systemic, but not subcutaneous vaccination.^72^ BCG^85C5^ vaccine induced marginally elevated levels of multifunctional cytokine-secreting cells (MFCs) in the lungs of mice compared with *wt-*BCG or BCG^85CFP^ vaccine (Fig. 7h). Likewise, it also enhanced the numbers of TB10.4^+^ CD8 T cells in the lungs (Fig. 7i). Since antigen-specific cytotoxic T cells lyse infected MΦs and their perforin can kill Mtb, their numbers may not necessarily reflect their function.^73^ Thus magnetic bead-purified CD8 T cells of lungs were evaluated for their ability to kill Mtb in MΦs using an in vitro cytotoxic-bactericidal assay. Figure 7i (CTL) indicates that lung-derived CD8 T cells of mice given BCG^85C5^ vaccine were more effective in killing Mtb than similar cells obtained from mice given *wt-*BCG or BCG^85CFP^ vaccine. Thus, vaccination with BCG^85C5^ vaccine induced qualitatively and quantitatively stronger T cells in the lungs of mice.

### BCG^85C5^ vaccine induces stronger T_EM_ and T_CM_ responses after aerosol “rechallenge” with Mtb

BCG vaccination does not protect adults against tuberculosis in developing countries, and even booster vaccination of BCG vaccinated children with MVA-expressing Ag85A failed to generate adequate protection against tuberculosis.^74^ In many viral infection models and Listeria infection, T_CM_ mediate long-term immunity.^65–67^ Since BCG^85C5^ vaccine induced a robust mix of T_EM_ and T_CM_ (Fig. 7e–g), we hypothesized that T_CM_ could persist longer and mount a stronger recall response, which could be ascertained through a mouse model that allowed the classic expansion, contraction, and recall of memory responses.^75^ In a recently described reinfection model of mouse tuberculosis, CD4 effector and central memory T cells generated by Mtb infection were soon lost after chemotherapy and bacteria returned.^76^ In a comparable study, TB10.4-specific CD8 T cells underwent a recall expansion after Mtb reinfection of mice, but the efficacy of protection against lung tuberculosis was not determined.^57^ Since these models evaluated “infection-induced” immunity against “reinfection”, we developed an improved model in which, the efficacy of “vaccination-induced” memory immunity was tested against infection followed by reinfection (Fig. 7a; rechallenge model). The hypothesis was that improved BCG^85C5^ vaccine, unlike Mtb infection, would induce T-cell populations that protect against reinfection.

**Fig. 8:**
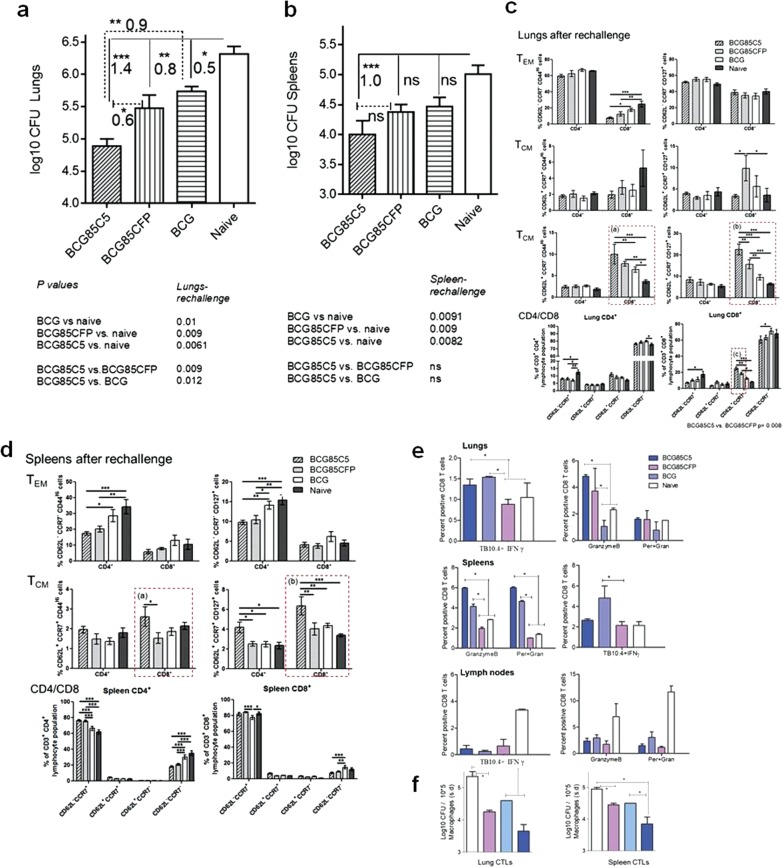
BCG^85C5^ vaccine induces a robust protection against rechallenge of tuberculosis in mice through expansion of effector (T_EM_) and central memory (T_CM_) T cells. **a**, **b** CFU counts of Mtb in the lungs and spleens after rechallenge (day 120). BCG^85C5^ reduces Mtb CFUs in the lung and spleens of mice after rechallenge better than wt-BCG. *P-*values and log10 difference of CFUs indicated (footnote; *n* = 5 mice per group; two-way ANOVA). **c**, **d** BCG^85C5^ induces both T_EM_ and T_CM_ T cells in the lungs and spleens after rechallenge (*p < 0.01; **p < 0.009; ***p < 0.007; one-way ANOVA). BCG^85C5^ induces more CD62L^+^ CD8 central memory T cells in the lungs and spleens compared with BCG^85CFP^ and *wt-*BCG (Fig. 7c, p boxes a–c; Fig. 7c, p boxes a, b). *wt-*BCG induced more CD62L^−^ CD4 effectors in the spleen (Fig. 7d). **e** CD8 T cells of organs were typed for TB10.4 tetramer-specific CD8 T cells and those expressing granzyme-B and perforin (three individual mice analyzed, and percent positive T cells averaged; *p < 0.0091; one-way ANOVA). BCG^85C5^ increases numbers of TB10.4^+^ CD8 T cells in the lungs and spleens correlating with an increase in expression of granzyme-B and perforin. **f** CD8 T cell pools purified from lungs and spleens were overlaid on Mtb infected macrophages for bactericidal activity as in Fig. 7i. BCG^85C5^ induced CD8 T cells show enhanced killing of intracellular Mtb (*p < 0.008; one-way ANOVA). *P-*values determined using one- or two-way ANOVA with Dunnett’s post-test

Figure 8a, b illustrates that, among vaccinated mice challenged with Mtb and cleared of organisms, rechallenge of Mtb infection was once again more effectively contained by the BCG^85C5^ vaccine, which was more effective than either BCG^85CFP^ or *wt-*BCG in the lungs (footnote, Fig. 8a, b). To determine the memory phenotype mediating this protection, T cells harvested from lungs and spleens were analyzed using flow cytometry.

*BCG*^*85C5*^ induces recall expansion of both CD4 and CD8 effectors and central memory T cells after reinfection. Figure 8c (p groups a, b, c*)* illustrates that vaccinated and unvaccinated mice showed a rapid expansion of CD4-T_EM_ and CD8-T_EM_ after rechallenge. However, BCG^85C5^ vaccinated mice had higher numbers of CD62^+^ CD8-T_CM_ in the lungs, compared with mice given BCG^85CFP^, *wt-*BCG or naive mice *(*p groups a, b, c; Fig. 8c). Furthermore, unlike the spleens after primary challenge (Fig. 2f), spleens of BCG^85C5^ vaccinated but rechallenged mice showed an increase in CD62L^+^ CD8-T_CM_ response (p groups a, b*;* Fig. 8d). Thus, protection induced by BCG^85C5^ against secondary aerosol challenge correlated with an expansion of both CD4 and CD8 effectors and an enhanced central memory T-cell response in the lungs and spleens. To further define the basis of long-term memory, T cells of mice rested after chemotherapy but before rechallenge were analyzed for intracellular expression of “T-box expressed in T cells” (T-bet) and Eomesodermin (Eomes), markers respectively of T_EM_ and T_CM_. The data show that BCG^85C5^ induced more Eomes^+^ T cells than either BCG^85CFP^or *wt*-BCG (Supplementary Fig. S13). This is consistent with the report that Eomes facilitates the emergence of long living T_CM_.^77^

BCG^85C5^ vaccine-induced CD8 T cells are functional after secondary challenge with Mtb. That CD4 T cells protect against tuberculosis by secreting T_H_1 cytokines is well established. Since the recall T-cell response to Mtb reinfection among vaccinated mice was associated with a stronger CD8 T-cell component (Fig. 8c, d), these cells were further analyzed using the TB10.4 tetramer and intracellular stains for granzyme-B and perforin followed by an in vitro cytotoxic-bactericidal assay.^57^ Figure 8e illustrates that the lungs and spleens of BCG^85C5^ and BCG^85CFP^ vaccinated mice contained increased numbers of TB10.4^+^IFNγ^+^ T cells compared with *wt-*BCG vaccinated or naive mice. Furthermore, BCG^85C5^ vaccinated mice contained more granzyme-B and perforin expressing CD8 T cells. Coincident with this observation, magnetic bead-purified CD8 T cells from lungs and spleens of BCG^85C5^ vaccinated mice had increased cytolytic and bactericidal activity in vitro and killed intracellular Mtb (Fig. 8f).

## Discussion

BCG is the most frequently used live attenuated (LAV) bacterial vaccine in humans, although it provides poor protection for adults against tuberculosis. BCG is unique inducing adult type T_H_1 immunity among neonates even when given at birth. In addition, BCG not only protects children against extra-pulmonary tuberculosis but also induces “trained” immunity, which protects children against multiple other infections.^78–80^ Thus there is a strong rationale to improve BCG vaccine for neonates, and some even recommend revaccination with BCG.^81^^,82^ In additional support, studies show that neonatal responses to BCG vaccine are sub-optimal. Neonates responded to antigens with more IL-10 which could be reversed via TLR stimulation restoring a robust pro-inflammatory profile and T_H_1 response to vaccines.^83–86^ Furthermore, infants produced weaker pro-inflammatory responses involving IL-12, TNF-α, and IL-β thereby enhancing susceptibility to tuberculosis.^87^ Polymorphisms in the TLR-1/2/6 pathway were also proposed to affect the efficacy of BCG since infants of tuberculosis endemic countries routinely received this vaccine.^88^^,89^

Despite being an LAV, BCG vaccine does not seem to induce long-lasting immunity, and indeed, it is perplexing that young adults who are otherwise immune competent are susceptible to tuberculosis in endemic areas. In our earlier studies, we sought to determine the molecular basis for the reduced immunogenicity of *wt*-BCG.^13,14,90,91^ LAVs are internalized into phagosomes which fuse with lysosome to generate peptides for the MHC-II-dependent activation of CD4 T cells. Since BCG evades PL fusion, we induced autophagy through either rapamycin pre-activation of DCs or mice or through hyperexpression of Ag85B that triggered autophagy in APCs, enhancing the efficacy of BCG^85^ vaccine.^14,20^ Three seminal studies including ours document that autophagy enhances MHC-II- and MHC-I-dependent antigen presentation enhancing respectively, CD4 and CD8 T-cell activation.^14^^,15,92^ Here, we expressed the Mtb-derived C5 autophagy-inducing peptide which also stimulated TLR-2 to culminate in the first autophagy-inducing recombinant BCG vaccine.

The discovery of autophagy-inducing and TLR-2 activating C5 peptide was fortuitous and highlighted the enigmatic observation that the immunodominant complex of ESAT-6 and CFP-10 contain both immune-suppressing and activating peptide motifs. While CFP-10-derived peptides were stimulatory, ESAT-6 contained both stimulatory and inhibitory motifs. Both Mtb and BCG secrete antigens, and it has been suggested that they bind and modulate APCs through their receptors. Indeed, ESAT-6 suppresses MΦs to IFN-γ,^25–27^ and induces suppressive IL-6 and TGF-β through TLR-2 signaling, thereby affecting cytosolic translocation of Mtb.^93,94^ Therefore during tuberculosis pathogenesis, ESAT-6 and CFP-10 complex perhaps skews immune responses beneficial to the pathogen.^27^ On the other hand, CFP-10 elicits robust response among humans and contained the autophagy-inducing and TLR-2 activating C5 motif.^95^ By expressing the C5 immunogenic moiety of Mtb in BCG^85B^, we markedly improved BCG vaccine as outlined below.

The immunogenicity of BCG vaccine, and its ability to activate CD4 T cells, depends upon three critical events; efficient processing of peptides in lysosomes, peptide epitope complexation with MHC-II, and their export to plasma membranes, where CD4 T cells can be activated in a milieu rich in pro-inflammatory T_H_1 cytokines. Interestingly, *wt*-BCG vaccine shows deficiencies related to these aspects.

Thus, BCG evades PL fusion^5^^,96^ and autophagy^97^, and we demonstrated that rapamycin-induced autophagy delivered BCG to lysosomes increasing antigen presentation in APCs and vaccine efficacy in mice.^14^ In this study, expression of C5 peptide in BCG^85C5^ brought out a novel effect of C5 peptide; it increased the TLR-2-dependent LC3 labeling of vaccine and its autophagy-dependent delivery to lysosomes (Fig. 5). Increased lysosomal delivery of BCG^85C5^ vaccine enhanced antigen presentation, consistent with the observation that TLR-2-mediated autophagy increases MHC-II-dependent antigen presentation.^98^ It is important to note here that BCG^85C5^ vaccine enhanced antigen presentation and T_H_1 cytokines in both MΦs and DCs. We showed earlier that rapamycin-activated DCs containing BCG were better than BCG alone in protecting mice against tuberculosis, thus highlighting the importance of DCs during vaccination.^99^ These observations are consistent with the observation that autophagy to increases both MHC-II- and MHC-I-dependent antigen presentation.^14^^,15^^,92^ Therefore, we propose the novel paradigm that C5 enabled BCG^85C5^ vaccine to overcome phagosome maturation block.

APCs present the lysosome-generated peptide epitopes through MHC-II to CD4 T cells expanding a critical arm of T_H_1 immunity. Paradoxically, both Mtb and BCG vaccine suppress the MHC-II expression in mouse and human APCs, and also desensitize the MΦ responses to IFN-γ.^43,48,100–102^ While a 19-kDa lipoprotein of BCG vaccine decreases MHC-II expression, our data show that C5 peptide overcomes the inhibitory effect and enhances MHC-II expression by reducing its ubiquitination and degradation (Fig. 4). This is consistent with our previous observation that commercial ligands for TLR-1/2 downregulate the MARCH1-ubiquitinating enzyme, thereby increasing the surface expression of MHC-II in APCs.^46^ Importantly, unlike previous studies, expression of C5 peptide in BCG^85C5^ vaccine bypassed the suppressive effects of BCG ligands like the 19-kDa lipoprotein and LAM.^43,103^ A second novel attribute of BCG^85C5^ vaccine is therefore a better MHC-II expression and antigen presentation.

*wt*-BCG induces a variety of cytokines in human monocyte–T cell co-cultures, but it is a poor inducer of IL-1β when given to humans.^104,105^ In addition, among neonates, it induces a poor pro-inflammatory cytokine response that could enhance susceptibility to tuberculosis. BCG^85C5^ vaccine induced TLR-2 activation and enhanced the secretion of IL-12, TNFα, and IL-1β (Figs 2, 3). Presumably, the induction of autophagy by BCG^85C5^ vaccine led to a better IL-1β response, since secretion of vesicles containing IL-1β is partly dependent on autophagy and furthermore, IL-12, TNF-α, and IL-1β self-regulate autophagy through a loop mechanism.^106^ Neonates have been reported to produce more IL-10 in response to antigens^83^^–^^86^ and show a weaker pro-inflammatory IL-12, TNF-α, and IL-β response enhancing susceptibility to tuberculosis.^87^ Thus, the third attribute of BCG^85C5^ vaccine is its ability to induce a robust IL-12, TNFα, and IL-1β response compared with *wt*-BCG (Figs 2, 3). It is relevant to note here that IL-12 induces T-bet enhancing effector function in T cells^107^^,108^; TNF-α mediates epigenetic modulation of T-helper lineage^109^, and IL-β regulates T-cell proliferation and helps to bypass tolerance.^110^

These mechanistic in vitro studies suggested that the BCG^85C5^ vaccine can elicit equally stronger and longer lasting T-cell response in mice. Figure 7, for example, shows that BCG^85C5^ induced strong antigen-specific CD4 (Ag85B-specific) and CD8 (TB10.4-specific) T cells among mice correlating with a better TLR-2-dependent protection against tuberculosis when compared with both *wt*-BCG and BCG^85^. Both these antigens are also strongly recognized by humans.^74,111^

An interesting aspect of BCG vaccination in mice is that it protects mice against primary challenge by about 1−log_10_ failing to eliminate Mtb, and furthermore poorly protects against reinfection. We addressed this defect by hypothesizing that robust immunity requires both a strong effector T-cell (T_EM_) response that generates short-term protection, and a critical central T-cell memory (T_CM_) response necessary for a long-term protection. It is relevant to recall here that many virus vaccines induce robust effector and central memory T-cell responses. Based on this logic, it was anticipated that modified vaccinia Ankara (MVA)-based vaccines expressing multiple Mtb-derived antigens should induce robust effector and central memory T cells. Although MVA-Ag85A followed by adjuvant-Ag85A booster did induce effector and central memory T cells in mice, to the best of our knowledge, a correlation between T_EM_ and T_CM_ to protection against tuberculosis has not been demonstrated.^112^ In this connection, it is important to note that *wt*-BCG induces a robust T_EM_ response, but a weaker T_CM_ response in mice.^113^

It is intriguing to observe that many viral vaccines can induce long-lasting immunity in humans although, few if any bacterial vaccines seem to induce long-lasting protection in humans. Using virus and *Listeria* infection models, Obar and Lefranquois proposed that both T_EM_ and T_CM_ cells are induced during the initial infection, although the T_CM_ cells are less prominent during initial stages, and become dominant over time with significant changes associated with the upregulation of the memory marker CD62L.^65–67^^,114^ They also proposed that the “strength“ of the initial interaction between APCs and T cells determines the T_CM_ response whereas, cytokines like IL-12, IL-7, and type I-IFNs affect the T-bet and Eomes transcription factors, which in turn regulate the expansion and maintenance of T_EM_ and T_CM_ lineages, respectively.^115^^,116^ Importantly, vaccine-induced APC-T cell interactions need to occur in an optimal environment with durability of antigen for long-term memory to emerge.^65–67^^,114,117,118^ Notably, “duration of contact with antigen” was thought to be critical for memory development.^118^ Since BCG^85C5^ induced strong antigen presentation both *i*n vitro (Figs 1–4) and in vivo (Fig. 7), we proposed that it would enable long-term memory in mice against tuberculosis. We noted that the yellow fever virus vaccine that induces long-term immunity activates multiple TLRs.^119^

An improved mouse model of vaccination illustrated that BCG^85C5^ expanded both T_EM_ and T_CM_ which correlated with a stronger protection against primary challenge as well as rechallenge with tuberculosis (Figs 7, 8). Importantly, the vaccine induced robust CD4 T cells against Ag85B, a major component of subunit vaccines against tuberculosis in humans and mice. In fact, a single dose of Ag85B vaccine elicited a CD4 T-cell response lasting a year in human volunteers.^120^ In addition a strong response of TB10.4-specific CD8 T cells were induced, and TB10.4-specific CD8 T cells were induced; this antigen is recognized among humans.^111^ Importantly, BCG^85C5^-induced CD8 T cells were functional against Mtb within MΦs.

It is known that T-bet induces T_EM_, while Eomes regulates T_CM_.^115,116^ BCG^85C5^ induced an expansion of T_EM_, contraction of this population during drug therapy, and allowed re-emergence after rechallenge; these were associated with a stronger expression, respectively, of T-bet and Eomes by the BCG^85C5^ vaccine (Fig. 8) (Supplementary Fig. S10). Thus, it was superior than *wt*-BCG by facilitating the transition of effector T-cell populations to longer-lasting central memory T cells as evident from the improved model of vaccination (Fig. 8a). To the best of our knowledge, BCG^85C5^ is unique in generating protection both against primary and rechallenge of tuberculosis in the mouse model.

The continued occurrence of active tuberculosis in BCG vaccinated population and the failure of certain subunit vaccines to boost BCG-induced immunity has led to varied attempts to improve tuberculosis vaccines. However, BCG is the only live attenuated safe vaccine that elicits adult-type Th1 immunity in neonates, and even generates substantial “trained” immunity that protects children against other infections.^121^ BCG continues to be a an essential vaccine in developing countries and many even recommend revaccination.^81,82^ This study generates optimism that it is feasible to design a recombinant BCG vaccine that enhances autophagy-dependent interactions between neonatal and adult APCs and T cells to generate long-lasting protection.

## Materials and methods

### MΦs and DCs

Primary bone marrow-derived MΦs (MΦs) and DCs from C57Bl/6 mice or TLR-2 KO mice (4–8 weeks old M/F, Harlan or Jackson, USA) were grown in Iscove’s medium with 10% FBS (IDM) and 10 ng/mL GM-CSF and CD11c beads (Miltenyi Inc, USA; 130-052-001) were used to deplete DCs from bone marrow cells cultured for 7 days. The CD11b + CD11c- MΦs or CD11c bead-purified CD11c + DCs were plated in GM-CSF-free medium and incubated overnight at 37 °C. They were then infected with *Mycobacterium bovis* BCG (Pasteur strain, ATCC 35734; MOI = 1), prepared as a single-cell suspension, for 4 h on a shaker at 37 °C washed and supernatants or cell lysates collected for further analysis. Viability of MΦ and DCs was >90% using trypan blue at the time of plating or at the end of experiments.

### Recombinant CFP-10 and ESAT-6 proteins and synthetic peptides

These were a kind gift from BEI, NIH. The overlapping peptides of both CFP-10 and ESAT-6 were synthesized to >90% purity by Genscript (USA). Thus, LPS-mediated TLR-4 activation was not an issue for the peptides.

### Construction, expression, and purification of ESAT-6 truncated proteins

ESAT-6 WT and the truncated proteins were obtained as previously described.^122,123^ Briefly, the full-length ESAT-6 gene (wt, residues 1–95) immediately followed by a C-terminal His6 tag was cloned into pET22b at the NdeI/XhoI sites. The DNA encoding C-terminal truncated ESAT-6 (residues 1-85, ΔC), which is immediately followed by a His6 tag, was obtained by PCR and cloned into pET22b at NdeI/XhoI sites. The DNA encoding the N-terminal truncated ESAT-6 (residues 11 -95, ΔN) and the DNA encoding both N- and C-terminal truncated ESAT-6 (residues 11-85, ΔN + C), both of which were followed by a C-terminal His6 tag, were obtained by PCR and cloned into pGEX4T-1 vector at BamHI/XhoI sites.

ESAT-6 wt and truncated proteins were expressed and purified as previously described.^122,123^ Briefly, pET22b-ESAT-6(WT) and pET22b-ESAT-6(ΔC) were expressed in BL21 (DE3) cells. The inclusion body was isolated and then solubilized in 8 M urea. The proteins were refolded on a nickel column and eluted with an imidazole gradient. The eluted proteins were further clarified by size exclusion chromatography to 90% purity. GST- ΔN and GST- ΔN + C were expressed as soluble proteins in BL21 (DE3) cells. The fusion proteins were purified on a glutathione–Sepharose 4B column. The purified GST fusion proteins were cleaved with thrombin, and His-tagged ΔN and ΔN + C were purified on a nickel column, followed by size exclusion chromatography.

### In vitro presentation Ag85B to BB7 CD4 T cells

MΦs and DCs were cultured in IDM and used as monolayers in 24-well plates for IL-2 assays. The Ag85B epitope (240–254; FQDAYNAAGGHNAVF)-specific T-cell hybridoma (BB7) was a kind gift of Drs. Cliff Harding and Henry Boom, CWRU, USA. Untreated or peptide activated APCs were infected with various BCG vaccine strains (MOI = 1) (washed and sonicated to obtain single cell CFU suspension) for 2 or 4 h followed by washing of monolayers with medium and over lay with T cells (20:1 ratio). Supernatants were collected 4 or 24 h later as indicated and tested for IL-2 using sandwich ELISA.

### Mycobacteria

Wild-type BCG (*wt-*BCG Pasteur) (ATCC #35734), Mtb Erdman (#35801) were from the American Type Culture Collection (ATCC), and were grown in Dubos’ broth and used afresh after three washes in PBS. Cultured mycobacteria were routinely >90% viable as evaluated by fluorescein diacetate stains (Invitrogen, USA). Recombinant BCG including *gfp-or rfp-*BCG were grown in kanamycin containing 7H9 broth. Single-cell suspensions for APC infection, aerosol infection, or vaccination of mice have been described earlier.^14^

### Western blot experiments

Six well plates were seeded with primary MΦs. They were activated with peptides as indicated for 4 h followed by addition of *wt-*BCG (MOI of 1). At time points, MΦs were washed thrice with ×1 PBS, and 200 µl 0.05% SDS/APM (anti-protease) mix was added to each well and incubated for 15 min. Lysates were then collected, and protein quantification (BCA, Pierce 23225) was performed. For western blot, 25 µg of the total protein was loaded per well of Bio-Rad criterion gels and transferred to PVDF membranes. Antibodies against both the native and phosphorylated proteins of c-Jun and CREB were used at a dilution of 1:500 (c-Jun (60A8), Cell Signaling #9165; ℗-c-Jun (Thr91), Cell Signaling #2303; CREB (48H2), Cell Signaling #9197; ℗-CREB (Ser133), Cell Signaling #9191). Secondary antibody was added (1:1000 α-rabbit, Cell Signaling #7074 s), and the membranes were then developed using ECL kit. Other inhibitors used were from EMD chemicals: 559388-SB 202190 p38 map kinase inhibitor II; 328006-ERK Inhibitor; 420119-JNK Inhibitor II; 217505, CBP-CREB Interaction Inhibitor. Western blot and densitometry analysis data are shown as mean band density normalized relative to β-actin or phosphorylated proteins (*n* = 3).

### Inhibition of TLR signaling in MΦs and DCs

Various inhibitors of mitogen-activated kinases (MAPK), AP-1/CREB were used to block signaling due to TLRs. TRAF-6 inhibitor peptide set and NF-kB inhibitor peptide set (#2004) were from Imgenex, USA, and IRAK1/4 inhibitor (#15409) was from Sigma (USA). AP-1 inhibitor (#SR11302) was from Tocris (USA). MΦs were treated with inhibitors for 2 h, activated with TLR-ligands for 2 h, and infected with BCG for 2 h prior to fixation and assay of surface MHC-II or antigen presentation. This rapid procedure was adapted to ensure >90% viability of MΦs.

### MHC-II assays

Surface staining for MHC-II and western blots for ubiquitinated MHC-II have been described in detail elsewhere.^46^ Briefly, primary MΦs or DCs were incubated with inhibitors of MAPK, AP-1, and CREB for 2 h, added with C5 peptide for 2 h, mixed with BCG for 2 h, and stained for MHC-II before analyzing using flow cytometry. Short-term activation and infection was used to prevent viability loss in this experiment and to minimize adherence of MΦs. Trypan blue staining showed >95% viability in MΦs and DCs after treatments.

### Autophagy experiments

MΦs and DCs were infected with either *rfp*- or *gfp*BCG (MOI = 1), with or without added peptides and after infection of 4 h with mixing, washed, and fixed for immunostains with antibodies against LC3, LAMP1, and CD68, as described.^14^ MΦs were either derived from C57Bl/6 or TLR-2 KO mice; RAW.A4 cells expressing *gfp*LC3 was prepared in the lab of Dr. Eissa, and has been published.^99^ Confocal images were acquired with a Nikon-N90 florescent microscope with NIS-Elements software that merges green and red fluorescence and computes colocalization and yields pixelated data 14 125. Visual scoring by an unbiased observer on blinded samples were compared with a computer-assisted image analysis using ImageJ software (http://rsbweb.nih.gov/ij/). To define colocalization, at least 50% or more of individual BCG bacilli were scored yellow. Percent colocalization was determined by counting gfp/rfp-BCG per MΦ in 50 separate MΦs in triplicates and averaging them for an experiment done twice on separate days. The colocalization scores shown in figures are for one of two similar experiments (mean ± SD).

### *P*-values for all non-mouse experiments

The data were analyzed using one-way analysis of variance (ANOVA) with Dunett’s post hoc test.

### Mouse tuberculosis protection assay

C57Bl/6 mice (4–6 weeks of age, M/F) were vaccinated with one dose of BCG (10^6^ CFU per dose) given alone or mixed with CFP-10 or ESAT-6-derived peptides (25 µg/mouse dose during first dose in Freund’s incomplete adjuvant). Mice were given a booster dose of the same peptide without adjuvant 2 weeks later. Mice were aerosol challenged with 100 CFU of Mtb H37Rv using a Glas-Col aerosol chamber (Terre Haute, IN), as described.^14^ Mice were rested for 4 weeks, and killed for lung CFU counts by plating lung homogenates on 7H11 agar plates. CFU counts were expressed as log10 per organ (four mice per time point per vaccine combination; *p-*values determined using one-way ANOVA).

### Construction of recombinant BCG (BCG^85C5^) that overexpresses fused Ag85B and C5 peptide

The BCG vaccine overexpressing, secreted Ag85B (BCG^85B^) has been described by us earlier.^14^ To prepare the new vaccine construct, a plasmid was first constructed that can express Ag85B and C5 peptide fusion and for this purpose, two oligonucleotide primers RV1886H and AG85BCFP20AA were synthesized. Primer RV1886H (5′-GCCACGGGATCCAATTCGTTGCGGTCCAAGATGGCGCCGTCT-3′) is based on the DNA sequences upstream to the coding region of the gene *Rv1886c* that codes for Ag85B of *M. tuberculosis*. A BamHI site was engineered in this primer. The primer AG85BCFP20AA (5′-AGTAGCGAATTCTCACTAGTCGAGTTCCTGCTTCTGCTTATTGGCTGCTTCTTGGAAGCGCACCACCGCGGCCTGGGCGCCGGCGCCTAACGAACTCTGCAGGTCACC-3′) is based on the 3′ sequence of *Rv1886c* and sequences for the 20_aa_ region (AQAAVVRFQEAANKQKQELD, representing C5 peptide) of the CFP-10 protein encoded by *Rv3874* of *M. tuberculosis*. This primer was engineered to have a unique EcoRI restriction site. These two primers were used in PCR to amplify a DNA region of approximately 1520 bp which has sequences of the promoter region of Rv1886c and coding sequences for full Ag85B protein and 20 AA peptide region from CFP-10 protein. The plasmid pSDRV18861 was used as a template for this reaction, which has full Rv1886c gene and its promoter region. The PCR amplified fragment was initially cloned into a pCR2.1 vector (Invitrogen). After confirming the sequences of the cloned fragment by DNA sequencing, the fragment was cleaved by cutting with BamHI and EcoRI, and subsequently cloned into pMV206 cut with similar enzymes. The resulting plasmid pSD85BC5 was transformed into wild-type BCG to create BCG^85BC5^ using standard protocols. Overexpression of Ag85B and C5 peptide was confirmed with immunoblot using antibodies against purified Ag85B and CFP-10 proteins of Mtb (both from BEI, NIH) (Supplementary Fig. S14). The ability to secrete Ag85B was measured using an antigen presentation assay described below. The presence of C5 was confirmed using T cells of C57Bl/6 mice immunized with CFP-10 protein given subcutaneously (two doses of 10 µg/dose each given 2 weeks apart) with Freund’s incomplete adjuvant. The T cells of these mice but not naive mice, elicited IFN-γ release when activated with MΦs containing BCG^85C5^ but not BCG^85B^ (Supplementary Fig. S10).

### Mouse vaccine experiments

The main mouse vaccine validation protocol is identical to the NIH mouse model and is illustrated in Fig. 7 and in the first half of Fig. 7a (up to 60 days). Two types of mouse vaccine models are shown in Fig. 7. Figure 7a tested wild-type C57Bl/6 mice and matching TLR-2-KO mice from Jackson Inc. Figure 8a used only *wt-*C57Bl/6 mice. Age- and sex-matched mice (6–8 weeks) were tested naïve or vaccines given s.c. to the hind legs of mice (estimated to contain 1 × 10^6^ CFUs through plating on 7H11 agar). After vaccination, at time points indicated, mice were aerosol challenged with approximately 100 CFU of virulent Mtb Erdman using a Glas-Col (Indiana, USA) aerosol apparatus. Four weeks after challenge or at indicated times, organs were harvested for CFUs as described before. Significance of differences in the CFU counts was calculated using two-way ANOVA as outlined below. Five mice per vaccine strain were used and three–four individual mice per time point for flow-cytometry analysis. Challenge Mtb was differentiated from BCG vaccine by culturing organ homogenates in 7H11 agar containing Thiophene-2- carboxylic acid hydrazide (10 µg/mL), which inhibits BCG, but not wild-type Mtb.

#### Post-vaccination follow-up

Mice were killed on day 21 post vaccination (three or four mice per group) and individually analyzed as follows.^56^ Splenocytes were stained using tetramers as described below. T cells from spleens were also stained for multifunctional cytokines after they were re-stimulated with a soluble protein antigen from Mtb Erdman as described before.^124^ Purified protein antigens used for recall immune responses (ESAT-6, CFP-10, antigen-85B were from BEI (NIH). IFN-γ assays were performed by stimulating splenocytes using specific protein antigens added at 1 µg/mL and 18 h supernatants assayed for IFN-γ using sandwich ELISA.

#### In vitro cytotoxic and bactericidal test of magnetic bead-purified CD8 T cells

The bone marrow-derived macrophages were infected with Mtb (MOI of 1) for 4 h and washed thoroughly to remove external bacteria. CD8 T cells were purified from the lungs or spleens of mice (*N* = 3), pooled and overlaid on Mtb-infected macrophages at a 1:100 ratio. Viability of macrophages using trypan blue was >90% after 72 h, at which time point, lysates of macrophages were plated for CFUs.

#### Drug-induced clearance

Isoniazid (INH) and rifampin were mixed in saline and mice given 10 mg/kg dose daily by gavage for 3 weeks following which mice were rested as indicated.^125^

#### *P*-value for CFU counts, five mice per time point

Mice were left untreated (five per group) or vaccinated with BCG followed 4 weeks later with aerosol challenge with Mtb. Mice were killed, and colony counts of Mtb measured in the lungs. The data are plotted as log_10_ CFU per organ per mouse. Allowing for a statistical power of 0.8–0.9, and a usual variance of 0.1–0.2 log_10_ CFU, a reduction in the mean CFU values between saline controls and test groups of about 0.6–0.7 log_10_ CFU is significant, when *n* = 5 animals are used (two-way ANOVA with Turkey’s post-hoc test used for *p-*values).

### Flow cytometry of lung T-cell numbers and memory T cells

T cells of the infected lungs post challenge were enumerated as per published procedures. Briefly, 3–4 mice per dose per group were killed, lungs, spleens, and lymph nodes were teased in IDM, T cells enriched and stained for CD4 and CD8 T cells in addition to surface receptors and intracellular IFN-γ followed by flow-cytometric analysis. The results were also reported as absolute numbers of T cells per organs after performing an initial organ cell count using trypan blue and staining and acquiring a fixed number of cells. (a) Lungs were processed with 1 mg/ml collagenase and 1 mg/ml elastase (Sigma Biologicals, USA) to break down the fibrous tissue material. It was then passed through cell strains and teased using frosted slides till a suspension of cells was obtained. The tissues were further treated with ACK lysis buffer (BioWhittaker, USA) to remove RBCs. Abs (CD4-FITC, CD8-FITC, IFNγ-APC, Perforin-APC, Granzyme-B-PE, T-bet-PE, Eomes-APC, PD1-APC, KLRG1-FITC, CD44-FITC, CD127-PE, CD62L-APC, CD3-APCCy7) (T-bet-PE, Eomes-APC, Perforin-APC, and Granzyme-B-PE) were purchased from Ebioscience, USA. Tetramers were obtained from NIH core facility, Emory University. Single-cell suspensions from spleens were stained with I-A(b) Mtb antigen-85B precursor (FQDAYNAAGGHNAVF) tetramer-APC/PE or with I-A(b) human class II-associated invariant chain peptide (PVSKMRMARPLLMQA) tetramer-APC as a negative control (NIH MHC Tetramer Core Facility at Emory University, Atlanta, GA), and were identified by flow cytometry. It should be noted that not all tetramers were compatible for C57Bl/6 genetic background mice. For phenotype analysis, we used a minimum number of 50 events in the tetramer gate, with a mean of 180 events. Flow staining was performed as per BD Biosciences protocol. Cell events were collected using Beckman-Coulter-Gallios cytometer, and the cytokine profile was analyzed using FlowJo software (Tree Star, Ashland, OR). The graphs were plotted and analyzed using graph pad prism software. The other tetramers used in this study were: H-2D(b) Mtb32A 309-318 GAPINSATAM (PE-Labeled Tetramer)^126^; H-2K(b) Mtb TB10.4 4-11 IMYNYPAM (PE-Labeled Tetramer)^127^; H-2D(b) *M. tuberculosis* PstS - SGVGNDLVL (APC-Labeled Tetramer).^128^ Of these one tetramer had not been tested earlier and the epitope was based in predictive algorithm (Immune epitope database); H-2D(b) *M.tuberculosis* ESAT-6 protein (aa17-25)—AIQGNVTSI (PE-Labeled Tetramer) (also available from ProImmune, USA).

All procedures including procedures involving animal or human cells were conducted in accordance with all relevant guidelines and procedures per approved IRB and Animal welfare committee (AWC) protocols of UT Health Sciences Center.

### Reporting summary

Further information on experimental design is available in the Nature Research Reporting Summary linked to this article.

## Supplementary information

Supplemental Information

Reporting Summary

## Data Availability

The data supporting the figures are available from the authors after approval of institutional IP-related regulations.

## Supplementary information

**Supplementary information** accompanies the paper on the *npj Vaccines* website (10.1038/s41541-019-0122-8).
