# Supplementary material for: An autophagy-inducing and TLR-2 activating BCG vaccine induces a robust protection against tuberculosis in mice
Source: NPJ Vaccines. 2019 Aug 5;4:34. doi: 10.1038/s41541-019-0122-8 (PMC6683161; doi:10.1038/s41541-019-0122-8)
Supplement: Supplementary file 1 — Supplemental Information [file 41541_2019_122_MOESM1_ESM.pdf]

Supplemental Information:

A recombinant BCG vaccine expressing an autophagy-inducing and TLR-2 activating peptide from *Mycobacterium tuberculosis* induces a robust immune responses in antigen presenting cells and enhances immunity against aerosol induced tuberculosis in mice. *Khan et al 2018*

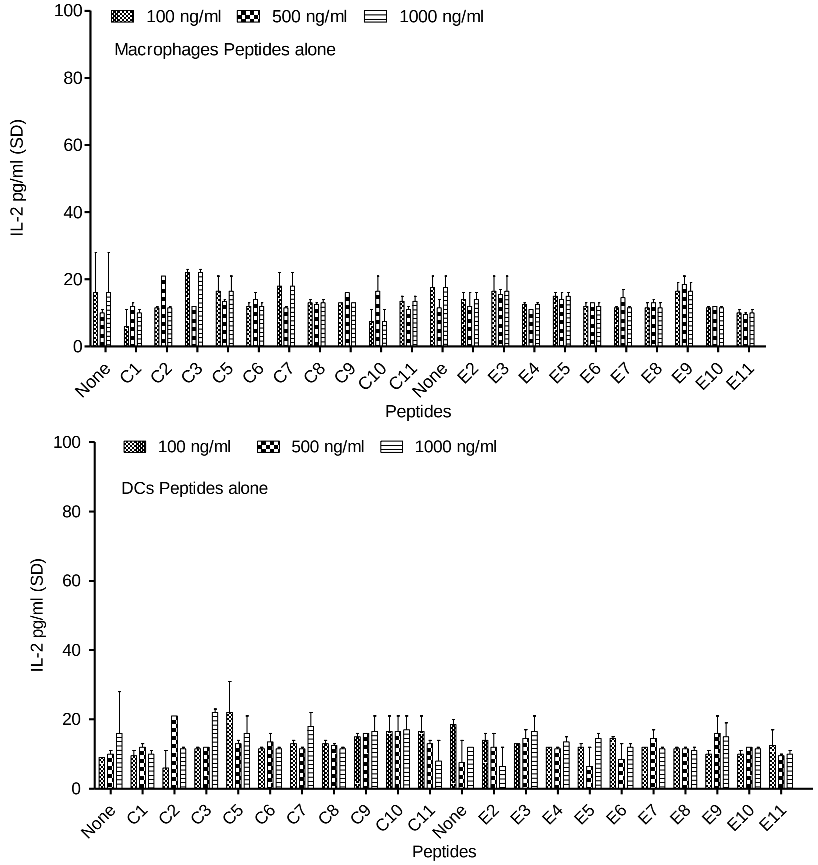

Figure-S1: The synthetic peptides from ESAT6 [E] and CFP10 [C] do not induce IL-2 responses on their own from either macrophages or DCs from C57Bl/6 mice.

Figure-S2: The Th1 cytokine stimulating motifs of ESAT6 [E] and CFP10 [C] for macrophages (M) and DCs (DC) are clustered around peptides E3 and E11 for ESAT-6 and around C5 for CFP-10. C57Bl/6 mouse bone marrow derived macrophages or DCs were stimulated with individual peptides (numbered), infected with BCG and cytokines determined 24 hr later using sandwich ELISA (* < 0.01; ** < 0.008; p values for peptide alone vs. peptide + BCG compared using one-way ANOVA with Dunett's post-hoc test).

Supplemental Information:

A recombinant BCG vaccine expressing an autophagy-inducing and TLR-2 activating peptide from *Mycobacterium tuberculosis* induces a robust immune responses in antigen presenting cells and enhances immunity against aerosol induced tuberculosis in mice. ***Khan et al 2018***

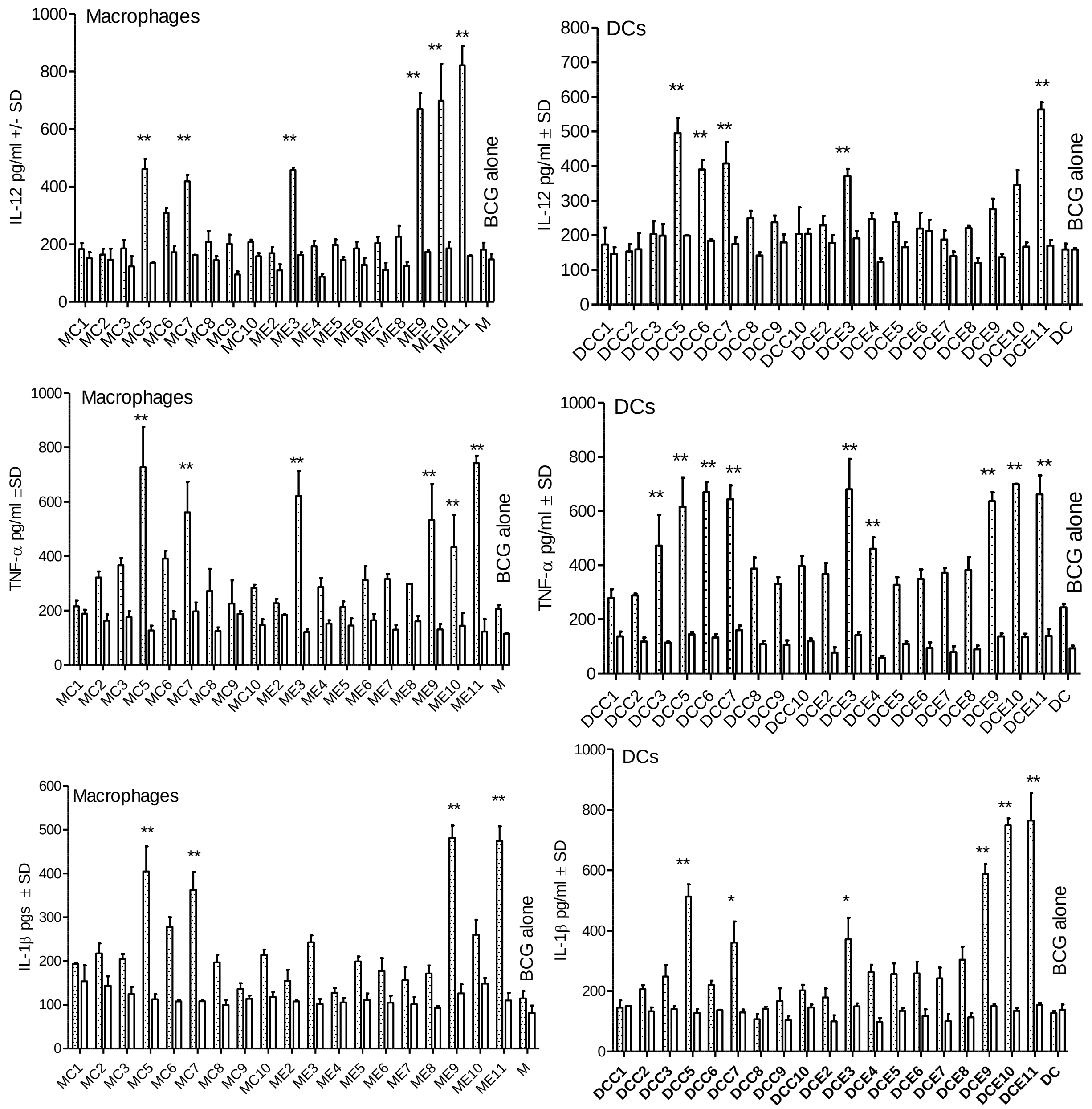

A recombinant BCG vaccine expressing an autophagy-inducing and TLR-2 activating peptide from *Mycobacterium tuberculosis* induces a robust immune responses in antigen presenting cells and enhances immunity against aerosol induced tuberculosis in mice. ***Khan et al 2018***

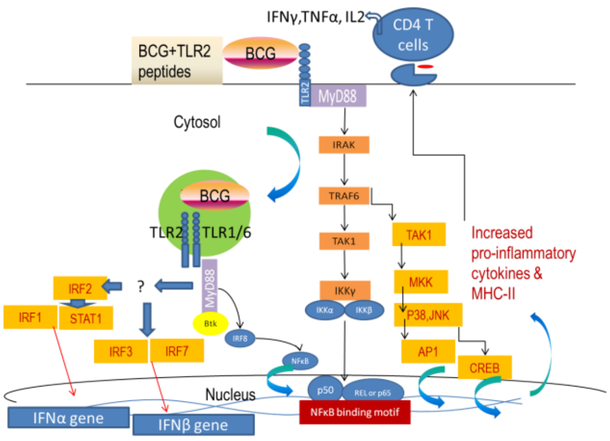

Figure-S3: Putative pathway of signaling by TLR-2 stimulating C5 peptide

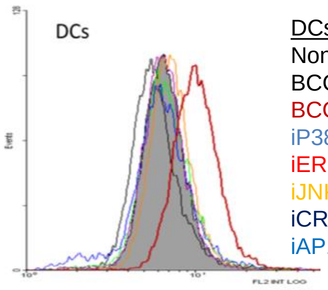

DCs	MFI
None	31 ± 3
BCG	40 + 3
BCG+C5	77 ± 6
iP38	38 ± 4
iERK	36 ± 3
iJNK	37 ± 4
iCREB	33 ± 3
iAP1	36 ± 2

Figure-S4: To determine if inhibitors of signaling cascade inhibited MHC-II expression (refer Fig.5, main text) on their own, DCs (macrophages show similar profile; not shown) were treated with inhibitors followed by flow cytometry for MHC-II. Positive controls of BCG and BCG+ C5 peptide treatments are shown.

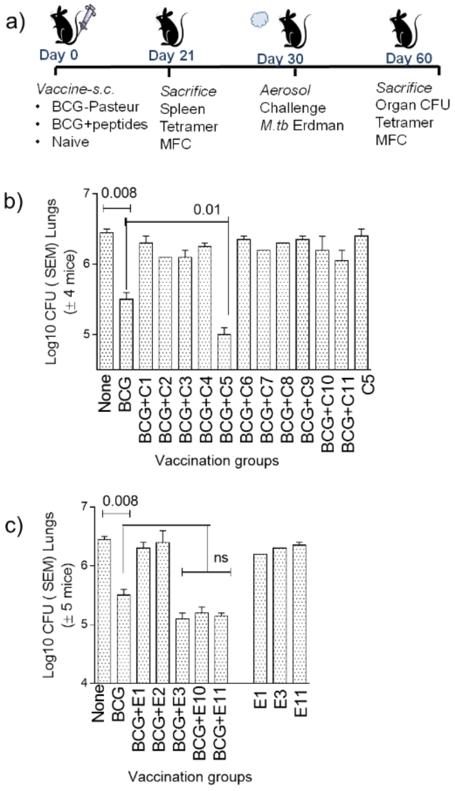

Figure-S5: CFP-10 and ESAT-6 derived peptides affect BCG vaccination against tuberculosis of mice in a differential manner. (a) Individual peptides of CFP-10 protein and selected ESAT-6 proteins (25 µg/dose; one s.c. dose) were combined with BCG (10^6CFU per dose) and used as vaccine against *M. tuberculosis* (H37Rv) in the mouse model. First dose of BCG + peptide vaccine was followed by a booster of peptide alone at 2 weeks. Mice were aerosol challenged with 100 CFU of M. tuberculosis (H37Rv; Mtb) and 4 weeks later mice were sacrificed for CFU counts. Lung organ homogenates were plated on 7H11 agar and CFUs counted after 4 weeks. (b-c). C5 peptide gives a better protection with BCG than other peptides of CFP-10 or ESAT6 derived peptides (n= 4 mice per group, two-way ANOVA with Dunett's post-hoc test).

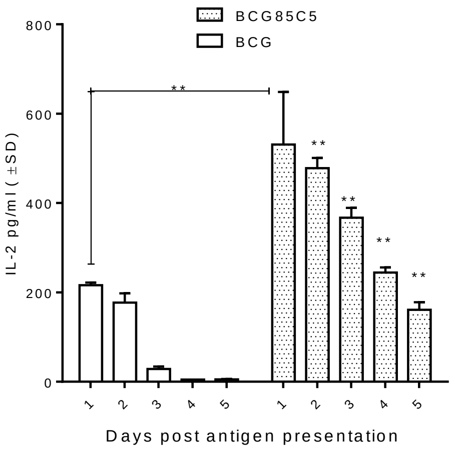

Figure-S6: **Sustained Antigen presentation is induced by BCG^{85C5} in DCs.** C57bl/6 mouse bone marrow derived DCs were infected with *wt*-BCG or BCG^{85C5} followed by antigen presentation using the Ag85B specific BB7 CD4 T cells (Methods). IL-2 was measured daily by aspirating 100 µL medium and replacing with 100 µL of fresh medium each day. BCG^{85C5} infected DCs show sustained antigen presentation over 5 5 days unlike *wt*-BCG (** $p < 0.009$ on day1 for groups compared; other p values were compared against wt-BCG induced IL-2 levels on respective days; one-way ANOVA with Dunett's post-hoc test).Similar profile is observed with mouse macrophages and for human THP1 macrophages overlaid with F4A9 cell line specific for Ag85B in the context of HLA-DR1 (not shown).

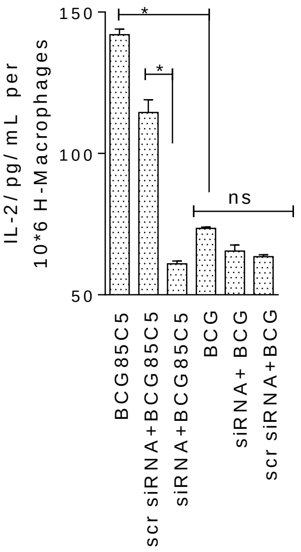

Figure-S7: **Antigen presentation induced by BCG^{85C5} in human macrophages is partially dependent on TLR-2.** Cord blood derived human macrophages (HLA-DR1) were plated in GM-CSF medium, rested and treated with siRNA vs. human TLR-2 (#TLR2 siRNA set; #i0006, Applied Biological Materials- ABM, USA) along with scrambled control. BCG strains as indicated were used for infection for 4 hr. followed by antigen presentation over night to HLA-DR1 compatible CD4 F4A9 T cell line (hybridoma) specific for an epitope of Ag85B (D. Canaday). IL-2 secreted was quantitated using sandwich ELISA. Knockdown of TLR-2 reduces antigen presentation by BCG^{85C5} but not BCG (* $p< 0.009$; one-way ANOVA with Dunett's post-hoc test). Note that human macrophages present antigen at lower rates compared to mouse macrophages.

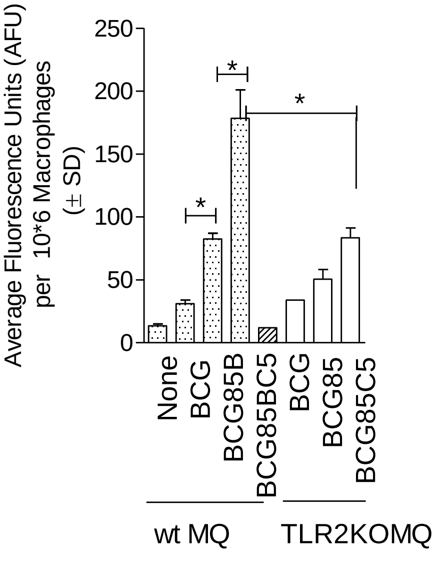

Figure-S8: **BCG^{85C5} candidate vaccine mediated TLR-2 activation enhances reactive oxygen species (ROS) response in macrophages.** C57Bl/6 or C57Bl/6 TLR-2KO mouse bone marrow derived macrophages were infected with BCG strains (MOI of 1) as indicated, washed and incubated. After 24 hr. the macrophages were read for average fluorescence units (AFUs ±SD) for triplicate wells using an Ascent-scan fluorimeter (485/530nm) after staining them with the vital fluorescent reagent dihydro-dichloro-fluorescein diacetate (DCFDA). BCG^{85C5} elicits a robust ROS response, which is reduced among TLR-2KO macrophages. Data of one of two similar experiments shown (* $p <0.07$; one-way ANOVA with Dunett's post-hoc test).

A recombinant BCG vaccine expressing an autophagy-inducing and TLR-2 activating peptide from *Mycobacterium tuberculosis* induces a robust immune responses in antigen presenting cells and enhances immunity against aerosol induced tuberculosis in mice. ***Khan et al 2018***

Figure-S9: **Antigen presentation induced by BCG^{85C5} is dependent on autophagy.** Wt-C57Bl/6 macrophages or those derived from mice with a conditional knockout of atg7 (that regulates autophagy) in DCs (*atg7-KODC*) were infected with BCG^{85C5} followed by antigen presentation to BB7 T CD4 T cells (Methods). Antigen presentations markedly decreased among atg7-KODCs. (* p< 0.007; one-way ANOVA with Dunett's post-hoc test). *Conditional KO atg7KO-DC mice produced by Jin Wang et al as described; Nat Med. 2014 May;20:503-10)*

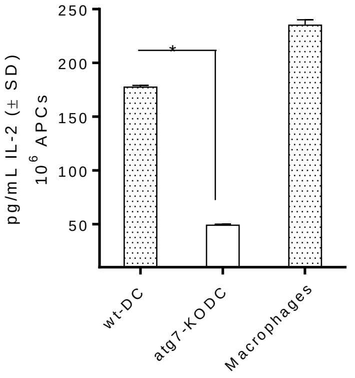

Figure-S10: **BCG^{85C5} vaccine elicits IFN-γ response from splenocytes of CFP-10 immunized mice but not wt-BCG.** C57Bl/6 mice immunized with CFP-10 protein given subcutaneously (2 doses of 10 µg/dose each given 2 weeks apart) with Freund's incomplete adjuvant. The splenocytes of mice were overlaid on macrophages infected (MOI=1) with either BCG or BCG^{85C5} and supernatants collected at 24 hr. tested for IFN-γ levels using sandwich ELISA (* p< 0.007; one-way ANOVA with Dunett's post-hoc test).Triplicate cultures of 10*6 macrophages were incubated with 10*6 splenocytes for each group.

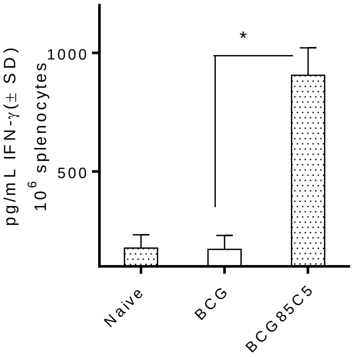

Figure-S11: Post aerosol challenge (Fig.6g-h) on day 60, lungs were stained for antigen specific T cells using tetramers for 4 antigens as cited (Methods). Mice vaccinated with BCG^{85C5} show better expansion of antigen specific T cells. Histogram from 1 mouse illustrated here and those form 3 mice averaged for Fig.6g-h.

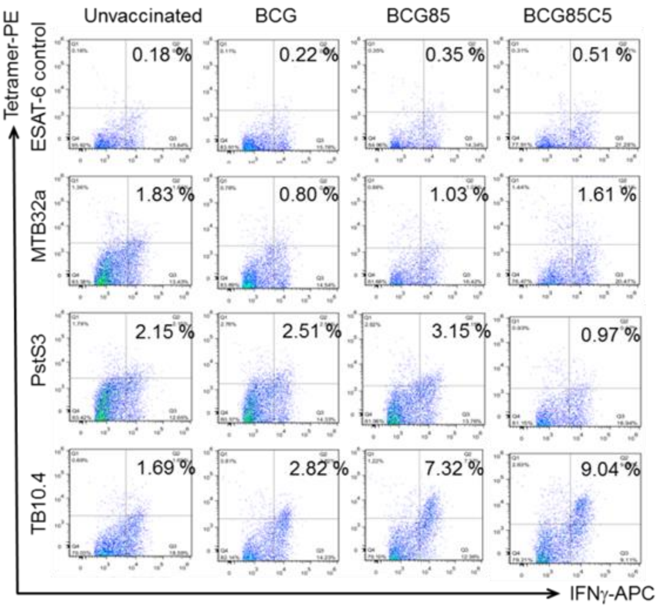

Figure-S12: Two weeks post vaccination (Fig.7a) spleens of mice vaccinated with BCG as shown, were tested for the presence of Ag85B specific CD4 T cells using the tetramer as cited in Methods (text). Mice vaccinated with BCG^{85C5} show the best expansion of CD4 T cells.

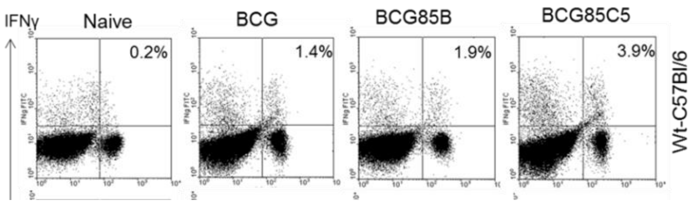

Figure-S13: The transcription factors T-bet and Eomesodermin (Eomes) regulate respectively effector and central memory lineage in CD8 T cells although their role in CD4 T cells remains unclear. T cells of mice rested after drug treatment (day 115) (Fig.8 main text) were tested for intracellular *T-bet* and *Eomes* positive T cells per organ. Data show that BCG[85C5] induces a stronger CD8+ Eomes response in both lungs and spleens compared to other vaccines. This is consistent with a robust recall of such T cells after rechallenge on day 120.

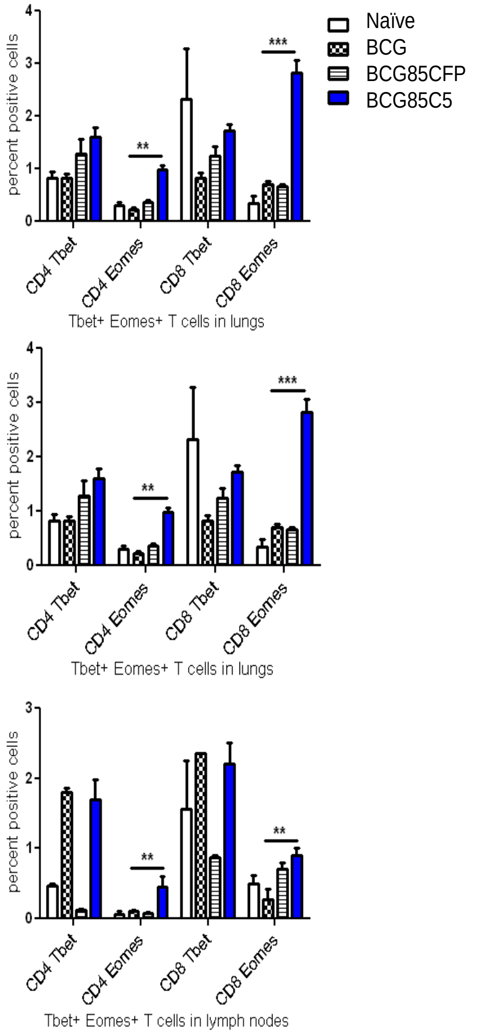

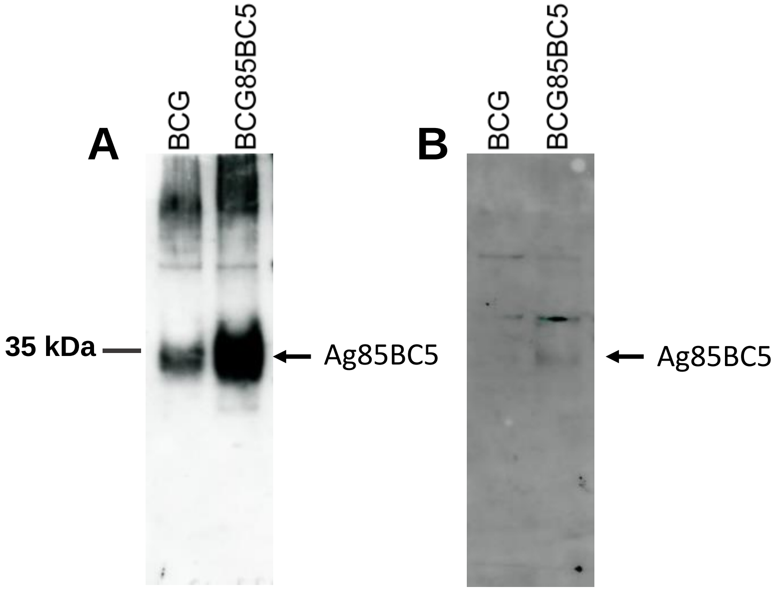

Supplemental Figure-14: Immunoblot demonstrating the expression of Ag85BC5 protein by recombinant BCG85C5 strain. Total protein extracts of from wild type BCG (**BCG**) and recombinant BCG85C5 (**BCG85C5**) strains were prepared. Approximately 25 µg of protein was separated on SDS-PAFE and transferred to Nitrocellulose membrane. The membrane was probed with antibodies against antigen 85B (α-Ag85B) and CFP-10 (α-CFP10) proteins. BCG85C5 stains showed several fold higher expression of Ag85C5 protein (**A**) and the C5 region could be detected by antibodies against CFP-10 (**B**).

A recombinant BCG vaccine expressing an autophagy-inducing and TLR-2 activating peptide from *Mycobacterium tuberculosis* induces a robust immune responses in antigen presenting cells and enhances immunity against aerosol induced tuberculosis in mice. ***Khan et al 2018***

Additional information

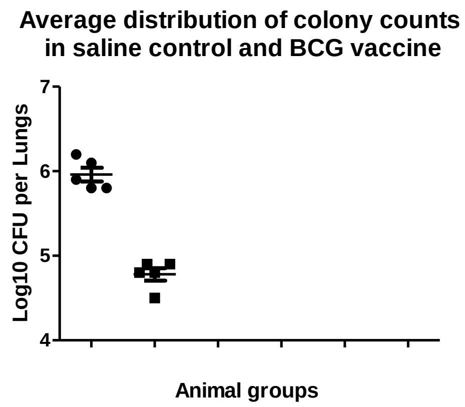

P value for CFU counts vaccine vs. untreated control, 5 mice per time point: Mice were left untreated (5 per group) or vaccinated with BCG followed 4 weeks later with aerosol challenge with MTB. Mice were sacrificed and colony counts of MTB measured in the lungs. Data are plotted as log_{10} CFU per organ per mouse. The example above shows that such early CFU data is highly discriminative and predictive of survival, and importantly the statistical power of these data is superior to survival data. Allowing for a statistical power of 0.8–0.9, and a usual variance of 0.1-0.2 log_{10} CFU, a reduction in the mean CFU values between saline controls and test groups of about 0.7 log_{10} CFU is significant, when n= 5 animals are used (2-way ANOVA used for p values).

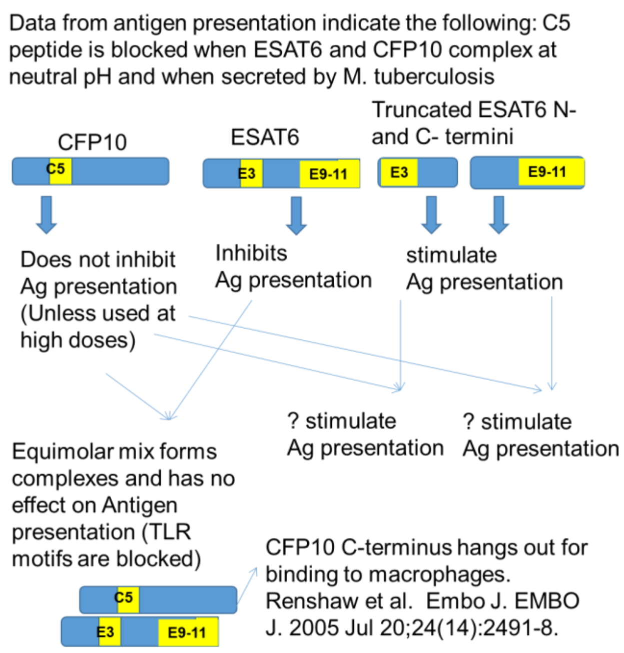

A tentative mechanism to explain why ESAT-6 and CFP-10 complex together and how it affects antigen presentation by APCs.
